# Studies on Chemical Composition of *Pueraria lobata* and Its Anti-Tumor Mechanism

**DOI:** 10.3390/molecules27217253

**Published:** 2022-10-26

**Authors:** Xiaoxue Fang, Yegang Zhang, Yiming Cao, Mengyao Shan, Dimeng Song, Chao Ye, Difu Zhu

**Affiliations:** 1 College of Pharmacy, Changchun University of Chinese Medicine, Changchun 130117, China; 2 Key Laboratory of Effective Components of Traditional Chinese Medicine, Changchun 130117, China; 3 College of Pharmacy, Jilin Medical University, Jilin 132013, China

**Keywords:** *Pueraria lobata*, colorectal cancer, cell cycle, network pharmacology, sandwicensin

## Abstract

Fourteen compounds were isolated from *Pueraria lobata* (Willd.) Ohwi by column chromatography and preparative thin-layer chromatography; the structures were identified by spectroscopic analysis and compared with data reported in the literature. Seven compounds were isolated and identified from *Pueraria lobata* for the first time: Linoleic acid, Sandwicensin, Isovanillin, Ethyl ferulate, Haginin A, Isopterofuran, 3′.7-Dihydroxyisoflavan. The other 10 compounds were structurally identified as follows: Lupenone, Lupeol, β-sitosterol, Genistein, Medicarpin, Coniferyl Aldehyde, Syringaldehyde. All compounds were evaluated for their ability to inhibit SW480 and SW620 cells using the CCK-8 method; compound **5** (Sandwicensin) had the best activity, and compounds **6**, **9**, **11** and **12** exhibited moderate inhibitory activity. In addition, the targets and signaling pathways of Sandwicensin treatment for CRC were mined using network pharmacology, and MAPK3, MTOR, CCND1 and CDK4 were found to be closely associated with Sandwicensin treatment for CRC; the GO and KEGG analysis showed that Sandwicensin may directly regulate the cycle, proliferation and apoptosis of CRC cells through cancer-related pathways.

## 1. Introduction

Colorectal cancer (CRC) is the second most common cause of cancer death and the third most common cancer worldwide [1]. CRC incidence and mortality rates have declined due to a gradual increase in cancer screening [2]. Although the combination of chemotherapy, radiotherapy and surgical resection can be used clinically to prolong the survival of patients, many patients still die from CRC every year, and many treatments are still associated with serious adverse effects and drug resistance, which reduce the quality of life and increase the psychological and economic burden of patients [3,4,5]. Therefore, it is essential to find adjuvant and less toxic alternative therapies that can improve the life quality of patients and prolong their life span. Chinese medicine, as a key adjunct to oncology treatment, can improve treatment efficacy, reduce drug resistance and prolong patient survival time [6].

*Pueraria lobata* is the dried root of *Pueraria lobata* (Willd.) Ohwi, one of the most important herbal medicines native to East Asia, South America and Europe [7]. *Pueraria lobata* has rich and wide pharmacological activities; Shiw et al. [8] found that *Pueraria lobata* significantly increased nitrogen monoxide (NO), cyclic guanosine monophosphate (cGMP) and endothelial nitric oxide synthase (eNOS) protein level expression and decreased angiotensin II type 1 receptor (AT1) and Caveolin-1 (CAV1) levels in spontaneously hypertensive rats, suggesting that *Pueraria lobata* has antihypertensive biological activity. In addition, *Pueraria lobata* can also reduce cholesterol (CH), triacylglyceride (TG) and low-density lipoprotein (LDL) levels in the blood of patients with hyperlipidemia; reduce lipid deposition; and improve hyperlipidemia [9]. Jearapongn et al. found that *Pueraria lobata* extract has the effect of increasing glutathione (GSH) and antioxidant activity [10]. Shuklar et al. mentioned that *Pueraria lobata* extract has the effect of inhibiting nephritis, and its main mechanism may be related to protein kinase C-α (PKC -α) and nuclear factor kappa-B (NF-κB) pathway downregulation, which exerts therapeutic effects by inhibiting the expression of inflammatory cytokines tumor necrosis factor-α (TNF-α), interleukin-6 (IL-6) and inducible nitric oxide synthase (iNOS) [11]. In addition, several studies have shown that *Pueraria lobata* has strong anti-tumor activity. Satpathys et al. used MTT to determine the toxic effects of aqueous extracts of *Pueraria lobata* synthesized with silver nanoparticles (AgNP3) on breast cancer (MCF-7 and MDA-MB-231) and ovarian cancer (SKOV-3); the results showed that AgNP3 had significant inhibitory effects in both cancers [12]. Zhang et al. found that puerarin-6″-O-xyloside had significant anti-tumor activity against CRC through induction of apoptosis [13]. Swaha Satpathy et al. demonstrated the significant cytotoxic effect of *Pueraria Mirifica* ethyl acetate extract (FRAC) on breast cancer (MCF-7 and MDA-MB-231) and ovarian cancer (SKOV-3) by in vitro experiments [14]. Although *Pueraria lobata* was found to have good anti-tumor activity in previous studies, the active components, and targets in *Pueraria lobata* for the treatment of CRC are not clear; therefore, the present study focused on the excavation of the anti-tumor chemical components of *Pueraria lobata* and clarification of their specific mechanisms of action. In this study, we isolated and purified the main active components of *Pueraria lobata* for anti-tumor treatment and obtained 14 compounds, among which compound **5** showed the best anti-tumor activity. In addition, we used network pharmacology to explore the anti-tumor targets as well as pathways of compound **5** and clarified the anti-tumor mechanism of *Pueraria lobata*, laying a good foundation for the development of anti-tumor drugs.

## 2. Results

### 2.1. Activity Screening of Different Fractions of Pueraria lobata

**Figure 1:**
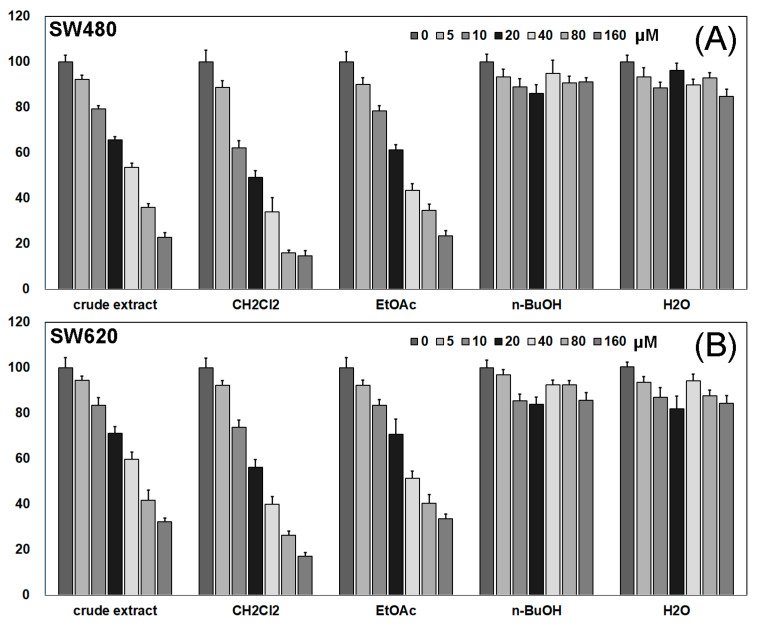
Cell viability as determined by CCK8 assay. (**A**) Different fractions of Pueraria lobata inhibit SW480 cell viability; (**B**) different fractions of Pueraria lobata inhibit SW620 cell viability.

To detect the toxic effects of Pueraria lobata extracts on SW480 and SW620 cells, we used the CCK-8 method to determine the growth of SW480 and SW620 cells after 48 h of the effects of different concentrations of Pueraria lobata extracts (0, 5, 10, 20, 40, 80, 160 μM). As shown in Figure 1A,B, CH_2_Cl_2_ and EtOAc extracts inhibited the growth of SW480 and SW620 cells in a dose-dependent manner, and n-BuOH and H_2_O extracts had no significant inhibitory effect; CH_2_Cl_2_ extracts showed more significant inhibitory activity compared with EtOAc extracts, indicating that the CH_2_Cl_2_ extract had a more pronounced inhibitory effect on CRC cells. Therefore, we isolated and purified the CH_2_Cl_2_ extract to further determine the material basis of Pueraria lobata for the treatment of CRC.

### 2.2. Structural Elucidation

Compound **1**: ^1^H-NMR (200 MHz, CDCl_3_), δ: 1.68 (s, 3H, H-30), 1.06 (s, 6H, H-24, H-26), 1.02 (s, 3H, H-23), 0.95 (s, 3H, H-27), 0.93 (s, 3H, H-25), 0.79 (s, 3H, H-28), 4.69 (1H, d, J = 2.3 Hz, H-29a), 4.57 (1H, d, J = 0.9 Hz, H-29b).^13^C-NMR (600 MHz, CDCl_3_), δ: 218.32 (C-3), 151 (C-20), 109.54 (C-29), 55.07 (C-5), 49.93 (C -9), 48.38 (C-18), 48.10 (C-19), 47.48 (C-4), 43.14 (C-14), 43.04 (C-17), 40.92 (C-8), 40.12 (C-22), 39.76 (C-2), 38.31 (C-13), 37.03 (C-10), 35.67 (C-16), 34.30 (C-1), 33.71 (C-7), 29.97 (C-21), 27.57 (C-15), 26.80 (C-23), 25.30 (C-12), 21.62 (C-11), 21.19 (C-24), 19.83 (C-6), 19.46 (C-30), 18.16 (C-28), 16.12 (C-25), 15.93 (C-26), 14.63 (C-27). Compound **1** was identified as Lupenone by comparison with the literature [15].

Compound **2**: ^1^H-NMR (300 MHz, CDCl_3_), δ: 0.76 (3H, s, H-23), 0.78 (3H, s, H-24), 0.82 (3H, s, H-27), 0.94 (3H, s, H-26), 0.96 (3H, s, H-25), 1.03 (3H, s, H-28), 1.68 (3H, s, H-30), 3.20 (1H, dd, J = 9.7, 4.7 Hz, H-3), 4.68 (1H, d, J = 2.3 Hz, H-29a), 4.56 (1H, dd, J = 2.4 Hz, H-29b).^13^C-NMR (600 MHz, CDCl_3_), δ: 109.5 (C-29), 151.1 (C-20), 79.16 (C-3), 55.45 (C-5), 50.59 (C-9), 48.45 (C-18), 48.13 (C-19), 43.15 (C-17), 42.98 (C-14), 40.98 (C-22), 40.15 (C-8), 39.01 (C-4), 38.86 (C-1), 38.20 (C-13), 37.32 (C-10), 35.74 (C-16), 34.43 (C-7), 29.85 (C-21), 28.14 (C-23), 27.60 (C-15), 27.56 (C-2), 25.29 (C-12), 21.08 (C-11), 19.46 (C-28), 18.47 (C-6), 18.16 (C-30), 16.27 (C-25), 16.13 (C-26), 15.52 (C-24), 14.70 (C-27). Compound **2** was identified as Lupeol after comparison with the literature [16].

Compound **3**: MS m/z: 279.26 [M+H]^−^.^1^H-NMR (300 MHz, CDCl_3_), δ: 5.35 (4H, m, J = 1.2 Hz, 9-H, 10-H, 12-H, 13-H), 2.77 (2H, m, J = 6.4 Hz, 11-H), 2.34 (2H, t, J = 7.0 Hz, 2-H), 2.04 (4H, m, J = 7.2 Hz, 8-H, 14-H), and 0.89 (3H, m, J = 3.6 Hz, 18-H). ^13^C-NMR (600 MHz, CDCl_3_), δ: 180.1 (C-1), 34.32 (C-2), 24.86 (C-3), 29.24 (C-4), 29.31 (C-5), 31.68 (C-6), 29.74 (C-7), 27.39 (C-8), 128.04 (C-9), 130.35 (C-10), 25.78 (C-11), 130.15 (C-12), 128.2 (C-13), 27.34 (C-14), 29.5 (C-15), 32.08 (C-16), 22.72 (C-17), 14.22 (C-18). Compound **3** was identified as Linoleic acid after comparison with the literature [17].

Compound **4**: ^1^H-NMR (300 MHz, CDCl_3_), δ: 5.35 (1H, d, J = 5.2 Hz, H-6), 3.52 (1H, m, H-3), 1.00 (3H, s, H-19), 0.92 (3H, d, J = 8.0 Hz, H-26), 0.90 (3H, d, J = 7.1 Hz, H-27), 0.85 (3H, d, J = 7.6 Hz, H-21), 0.80 (3H, t, J = 7.9 Hz, H-29), and 0.68 (3H, s, H-18). ^13^C-NMR (600 MHz, CDCl_3_), δ: 140.89 (C-5), 121.86 (C-6), 71.95 (C-3), 56.91 (C-14), 56.2 (C-17), 50.27 (C-9), 45.98 (C-24), 42.46 (C-4), 42.43 (C-13), 39.92 (C-12), 37.4 (C-1), 36.65 (C-10), 36.29 (C-20), 34.09 (C-22), 32.06 (C-7), 32.05 (C-8), 31.79 (C-2), 29.29 (C-23), 28.39 (C-16), 26.22 (C-25), 24.45 (C-15), 23.21 (C-28), 21.23 (C-11), 19.97 (C-27), 19.54 (C-19), 19.18 (C-21), 18.93 (C-26), 12.13 (C-18), 12.0 (C-29). Compound **4** was identified as β-sitosterol after comparison with the literature [18].

Compound **5**: MS m/z: 337.53 [M+H]^−^.^1^H-NMR (300 MHz, CDCl_3_), δ: 1.65 (3H, s, H-16), 1.75 (3H, s, H-15), 3.28 (2H, t, J = 6.8 Hz, H-12), 3.52 (1H, m, H-6a), 3.62 (1H, dd, J = 30.4, 19.5 Hz, H 6-α), 3.80 (3H, s, OCH3-H), 4.23 (1H, dd, J = 10.9, 5.0 Hz, H-6-β), 5.23 (1H, t, J = 7.3, H-13), 5.45 (lH, d, J = 6.8 Hz, H-11a), 6.40 (1H, d, J = 3.6 Hz, H-4), 6.41 ( 1H, d, J = 2.0 Hz, H-2), 6.56 (1H, d, J = 2.5 Hz, H-8), 7.01 (1H, d, J = 8.1 Hz, H-7), 7.41 (1H, d, J = 8.4 Hz, H-1). ^13^C-NMR (600 MHz, CDCl_3_), δ: 132.56 (C-1), 113.44 (C-1a or C-10), 109.71 (C-2), 158.7 (C-3 or C-10a), 103.15 (C-4), 156.77 (C-4a or C-9), 66.65 (C-6), 40.12 (C-6a), 119.41 (C-6b), 121.69 (C-7), 103.68 (C-8), 156.98 (C-9 or C-4a), 113.29 (C-10 or C-1a), 158.65 (C-10a or C-3), 77.98 (C-11a), 23.02 (C-12), 122.39 (C-13), 131.65 (C-14), 25.95 (C-15), 17.88 (C-16), 56.07 (OCH3). Compound **5** was identified as Sandwicensin after comparison with the literature [19,20]. 

Compound **6**: MS m/z: 269.07 [M+H]^−^.^1^H-NMR (300 MHz, CDCl_3_), δ: 7.02 (1H, s, H-2), 6.92 (2H, d, J = 8.3 Hz, H-2′, H-6′), 5.59 (2H, d, J = 8.3 Hz, H-3′, H-5′), 6.51 (1H, d, J = 8.3 Hz, H-8), 5.71 (1H, d, J = 8.3 Hz, H-6). ^13^C-NMR (600 MHz, CDCl_3_), δ: 185.91 (C-4), 163.52 (C-7), 161.26 (C-4′), 156.52 (C-9), 156.39 (C-5), 151.88 (C-2), 130.94 (C-2′, C-6′), 122.2 (C-1′), 121.14 (C-3), 116.42 (C-3′, C-5′), 105.22 (C-10), 100.72 (C-8), 91.85 (C-6). Compound **6** was identified as Senistein by comparison with the literature [21].

Compound **7**: MS m/z: 153.06 [M+H]^−^.^1^H-NMR (300 MHz, CDCl_3_), δ: 9.80 (1H, s, -CHO), 7.41 (1H, dd, J = 1.5, 5.4 Hz, H-6), 7.40 (1H, d, J = 1.0 Hz, H-2), 7.02 (1H, d, J = 8.5 Hz, H-5), 3.94 (3H, s, -OCH3). ^13^C-NMR (600 MHz, CDCl_3_), 191.19 (-CHO), 129.83 (C-1), 108.95 (C-2), 152.02 (C-3), 147.41 (C-4), 114.61 (C-5), 127.7 (C-6), 56.18 (-OCH3). Compound **7** was identified as Isovanillin after comparison with the literature [22].

Compound **8**: MS m/z: 221.03 [M+H]^−^.^1^H-NMR (300 MHz, CDCl_3_), δ: 7.61 (1H, d, J = 15.9 Hz, H-3), 7.07 (1H, dd, J = 8.1, 1.7 Hz, H-9), 7.03 (1H, d, J = 1.3 Hz, H-5), 6.92 (1H, d, J = 8.1 Hz, H-8), 6.29 (1H, d, J = 15.9 Hz, H-2), 4.26 (2H, q, J = 7.1 Hz, H-10), 3.93 (3H, s, H-12), 1.33 (3H, t, J = 7.1 Hz, H-11). ^13^C-NMR (600 MHz, CDCl_3_), δ: 167.44 (C-1), 115.76 (C-2), 144.81 (C-3), 127.15 (C-4), 109.38 (C-5), 146.87 (C-6), 148.03 (C-7), 114.82 (C-8), 123.12 (C-9), 60.52 (C-10), 14.51 (C-11), 56.06 (C-12). Compound **8** was identified as Ethyl Ferulate by comparison with the literature [23].

Compound **9**: MS m/z: 269.18 [M+H]^−^.^1^H-NMR (300 MHz, CDCl_3_), δ: 3.56–3.47 (1H, m, H-6a), 3.63 (1H, t, J = 10.9 Hz, H-6-a), 3.79 (3H, s, H-12), 4.24 (1H, dd, J = 10.9, 5.0 Hz, H-6-β), 5.51 ( 1H, d, J = 6.8 Hz, H-11a), 6.36 (1H, d, J = 4.8 Hz, H-10), 6.49–6.37 (2H, m, H-4, H-8), 6.64 (1H, dd, J = 8.5, 2.2 Hz, H-2), 7.07 (1H, d, J = 8.6 Hz, H-7), 7.42 (1H, d, J = 8.5 Hz, H-1). ^13^C-NMR (600 MHz, CDCl_3_), δ: 131.99 (C-1), 112.43 (C-1a), 109.32 (C-2), 157.13 (C-3), 101.78 (C-4), 156.69 (C-4a), 66.71 (C-6), 39.58 (C-6a), 125.1 (C-7), 119.5 (C-7a), 107.83 (C-8), 161.12 (C-9), 98.52 (C-10), 160.77 (C-10a), 78.79 (C-11a), 55.52 (C-12). Compound **9** was identified as Medicarpin by comparison with the literature [24].

Compound **10**: MS m/z: 177.01 [M+H]^−^.^1^H-NMR (300 MHz, CDCl_3_), δ: 9.65 (1H, d, J = 7.7 Hz, H-9), 7.40 (1H, d, J = 15.8 Hz, H-7), 7.12 (1H, dd, J = 8.1, 1.8 Hz, H-6), 7.07 (1H, d, J = 1.6 Hz, H-2), 6.96 (1H, d, J = 8.2 Hz, H-5), 6.59 (1H, dd, J = 15.8, 7.8 Hz, H-8), 3.95 (s, 3H, OCH3-H). ^13^C-NMR (600 MHz, CDCl_3_), δ: 193.75 (C-9), 153.22 (C-7), 149.12 (C-4), 147.11 (C-3), 126.79 (C-1), 126.57 (C-8), 124.20 (C-6), 115.09 (C-5), 109.60 (C-2), 56.15 (OCH3-C). Compound **10** was identified as Coniferyl aldehyde by comparison with the literature [25].

Compound **11**: MS m/z: 299.10 [M+H]^−^.^1^H-NMR (300 MHz, CDCl_3_), δ: 6.94 (2H, dd, J = 8.6 Hz, J = 6.1Hz, H-5, H-6′), 6.72 (1H, d, J = 8.5 Hz, H-5′), 6.54 (1H, s, H-4), 6.42 (2H, m, H-6, H-8), 5.00 (1H, d, J = 0.6 Hz, H-2), 3.94 (s, 3H, OCH3-H), 3.81 (s, 3H, OCH3-H). Compound **11** was identified as Haginin A after comparison with the literature [26].

Compound **12**: MS m/z: 285.08 [M+H]^−^.^1^H-NMR (300 MHz, CDCl_3_), δ: 7.60 (1H, d, J = 8.7 Hz, H-6′), 7.40 (1H, d, J = 8.3 Hz, H-4), 7.11 (1H, s, H-3), 6.99 (1H, d, J = 1.4 Hz, H-7), 6.82 (1H, d, J= 8.7 Hz, H-5′), 6.76 (1H, dd, J = 8.3, 2.1 Hz, H-5), 3.99 (2′-OCH3), 3.93 (3′-OCH3). Compound **12** was identified as Isopterofuran after comparison with the literature [27].

Compound **13**: MS m/z: 241.09 [M+H]^−^.^1^H-NMR (300 MHz, CDCl_3_), δ: 2.92 (d, J = 8.4 Hz, 2H, H-7), 3.15 (d, 1H, J = 7.9 Hz, H-8), 3.96 (d, J = 12.9 Hz, 1H, H-9a), 4.29 (dd, J = 13.0, 9.8 Hz, 1H, H 9b), 6.40 (m, 2H, H-2, H-4), 6.61–7.01 (m, 4H, H-11, H-12, H-13, H-15), 7.04–7.23 (m, 1H, H-1). Compound **13** was identified as 3′7-Dihydroxyisoflavan after comparison with the literature [28].

Compound **14**: MS m/z: 181.31 [M+H]^−^.^1^H-NMR (300 MHz, CDCl_3_), δ: 9.82 (1H, s, CHO), 7.15 (2H, s, H-2, 6), 3.82 (6H, s, OCH3). ^13^C-NMR (400 MHz, CDCl_3_), δ: 190.92 (CHO), 147.46 (C-3, 5), 140.88 (C-4), 128.54 (C-1), 107.57 (C-2, 6), 56.65 (OCH_3_). Compound **14** was identified as Syringaldehyde by comparison with the literature [29].

### 2.3. Activity Screening of Compounds ***1***–***14***

**Figure 2:**
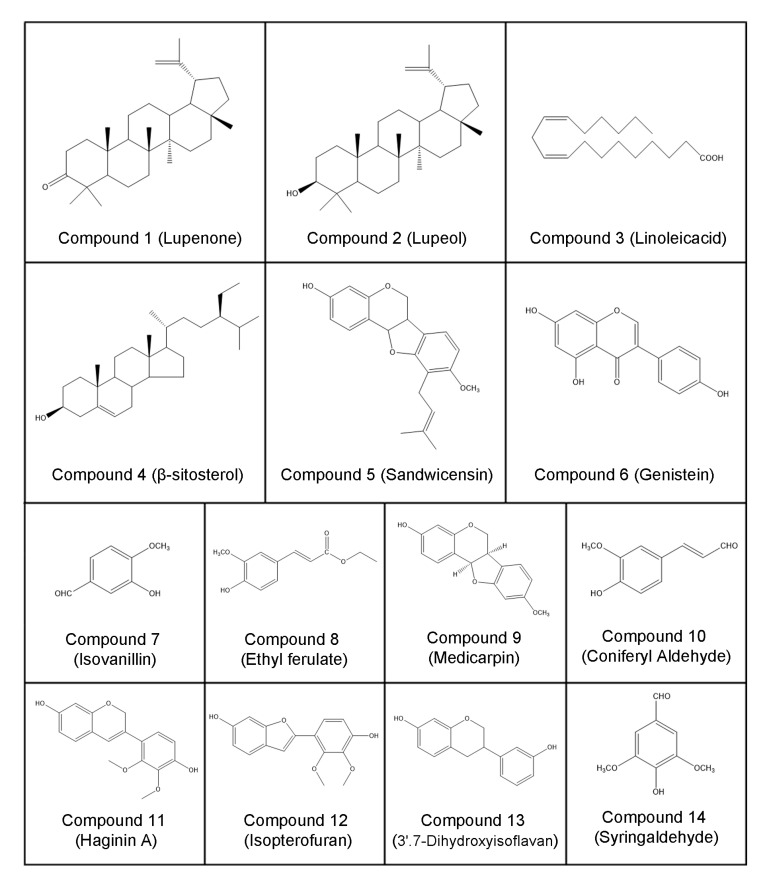
Structure of compounds **1**–**14**.

**Figure 3:**
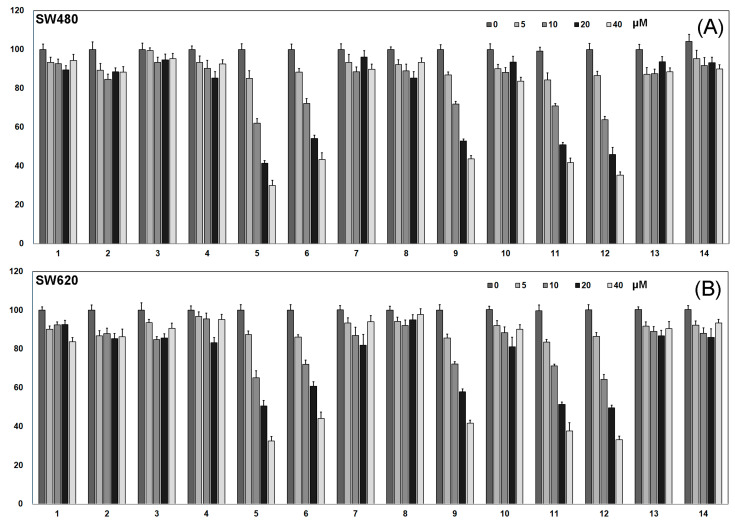
Cell viability as determined by CCK8 assay. (**A**) Compounds **1**–**14** inhibit SW480 cell viability; (**B**) Compounds **1**–**14** inhibit SW620 cell viability.

To further determine the material basis of *Pueraria lobata* for the treatment of CRC, we isolated and purified CH_2_Cl_2_ extracts to obtain compounds **1**–**14,** as shown in Figure 2. The effects of compounds **1**-**14** on the growth of SW480 and SW620 cells were determined by the CCK-8 method. As shown in Figure 3A,B, compounds **5**, **6**, **9**, **11**, **12** showed dose-dependent inhibition of SW480 and SW620 cell growth, while the other compounds had no significant inhibitory effect. The effect of compound **5** was the most obvious among these compounds, indicating that this compound is the main active ingredient of *Pueraria lobata* in the treatment of CRC.

### 2.4. Target Screening of Sandwicensin in the Treatment of CRC and Construction of Its Network Relationship

**Figure 4:**
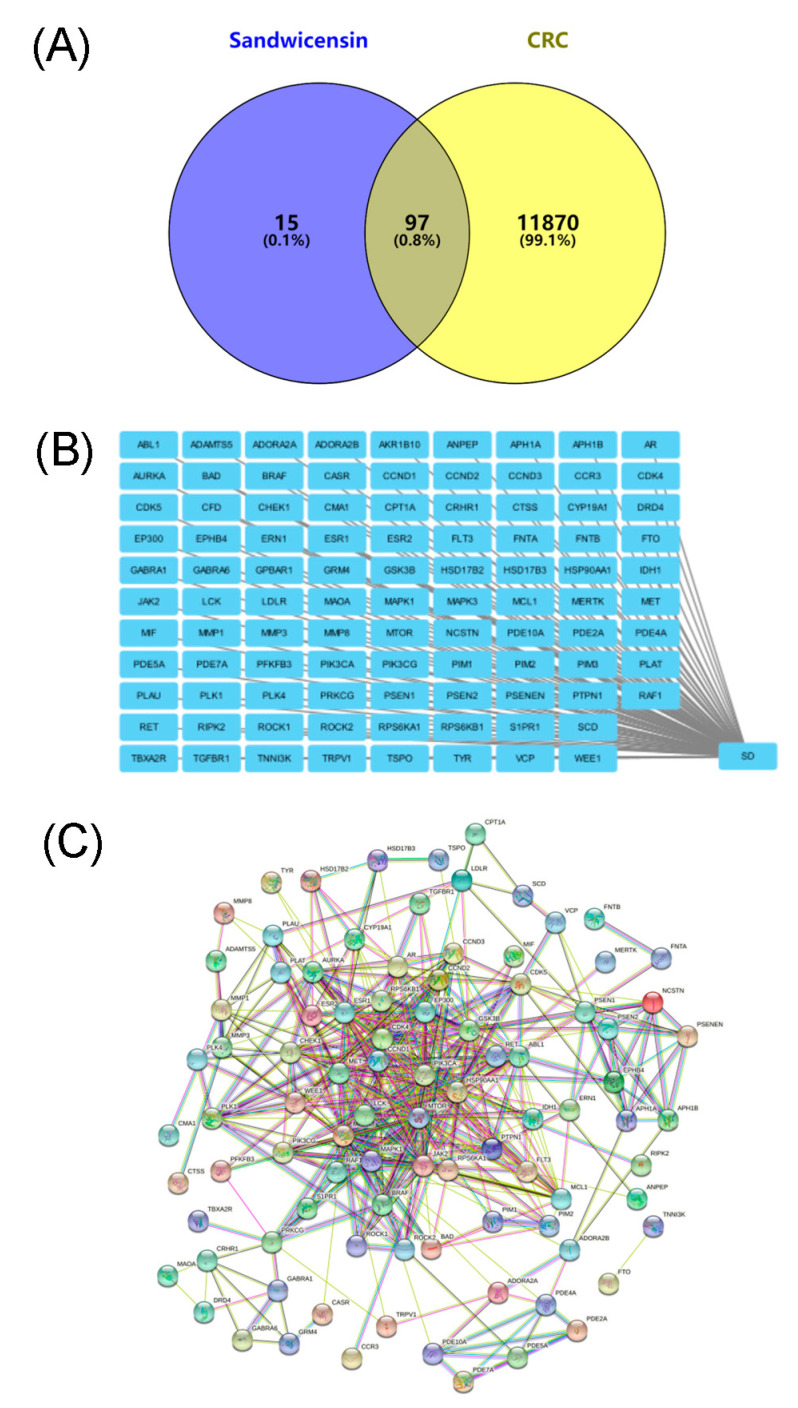
Network pharmacology analysis. (**A**) Venn diagram; (**B**) network of the treatment of CRC with Sandwicensin; (**C**) PPI network analysis. Nodes represent target proteins and edges represent interactions between target proteins.

In order to clarify the mechanism of Sandwicensin in the treatment of CRC, we performed target prediction as well as pathway analysis using network pharmacology; no target was found after searching “Sandwicensin” using the TCMSP database, and 112 predicted targets of “Sandwicensin” were retrieved from the Swiss Target Prediction database. A total of 11,967 disease targets were collected in five databases: DisGeNET, GeneCards, OMIM, TTD and Drugbank. Disease and component targets were mapped and a Venn diagram was drawn, as shown in Figure 4A, with 97 disease and component common targets. The consensus targets were visualized by using Cytoscape 3.2.1 software (http://www.cytoscape.org, accessed on 20 September 2022) to draw a component–target network map. As shown in Figure 4B, it includes 1 component node and 97 disease-related target nodes.

### 2.5. PPI Network Analysis of Target Proteins of Sandwicensin in CRC

The PPI network was constructed by uploading the targets of Sandwicensin for CRC in the String database and excluding the free nodes. As shown in Figure 4C, the PPI network included 93 nodes with 470 edges. The 93 target proteins were imported into Cytoscape 3.2.1 software (http://www.cytoscape.org, accessed on 20 September 2022) for network analysis and then sorted according to the degree values, as shown in Table S1. Targets with larger degree values were targets of Sandwicensin for CRC, such as MAPK3, MTOR, HSP90AA1, CCND1 and MAPK1. Table S2 lists the pairs of interacting targets with high confidence scores (confidence scores > 0.95 are considered high), such as AKT1 and NOS3, HIF-1α and STAT3, FOS and JUN, MMP2 and TIMP2, and AKT1 and PIK3CA, suggesting that the interaction of these targets may have a key role in the treatment of CRC with Sandwicensin.

### 2.6. GO Annotation and KEGG Pathway Enrichment Analysis

**Figure 5:**
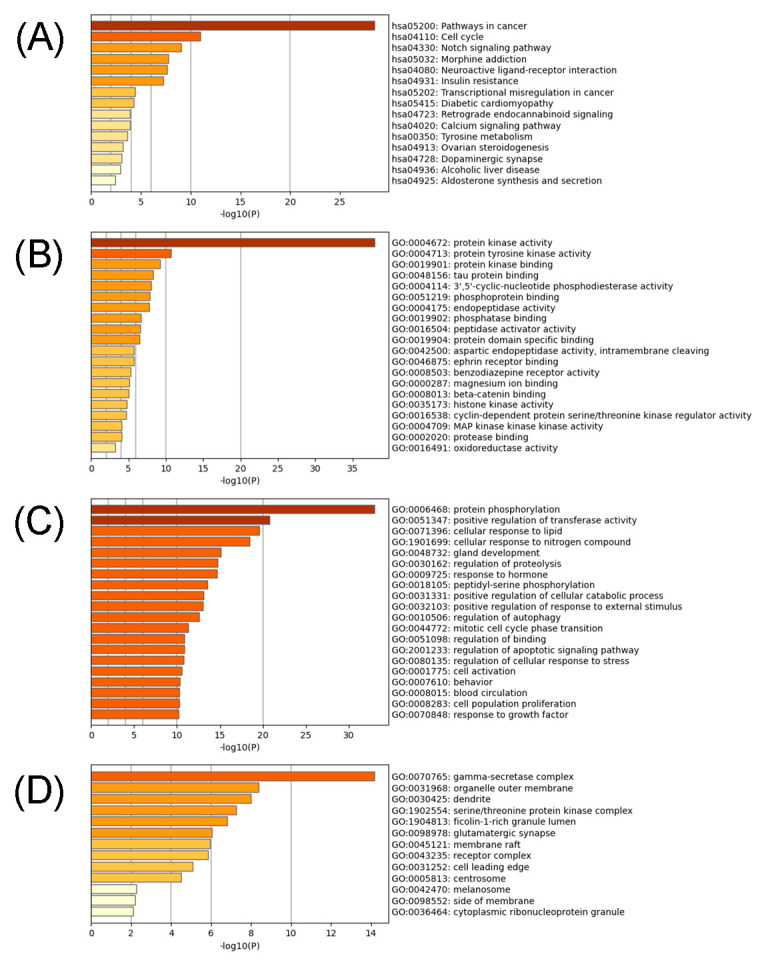
GO annotation and KEGG pathway enrichment analysis. (**A**) KEGG pathway analysis; (**B**) GO molecular function analysis; (**C**) GO biological process analysis; (**D**) GO cellular component analysis.

**Figure 6:**
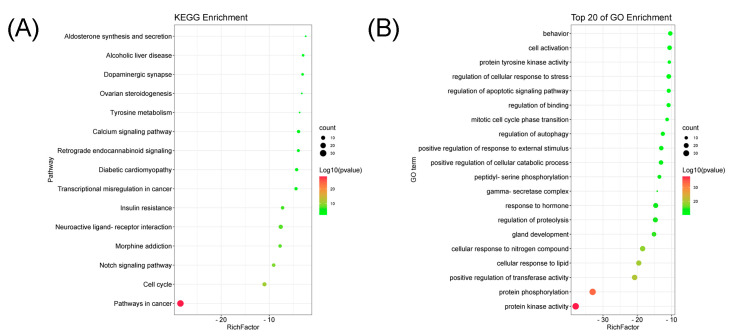
Bubble chart. (**A**) KEGG pathway enrichment analysis; (**B**) GO annotation analysis.

To further elucidate the functional process of Sandwicensin for the treatment of CRC, the three parts, namely biological process (BP), cellular component (CC) and molecular function (MF), of the intersecting targets were analyzed using GO enrichment analysis. The results showed that a total of 53 enrichment results were obtained. We selected the top 20 biological functions (*p* < 0.05), as shown in Table S3 and Figure 5B–D and Figure 6B. Through the enrichment analysis of the intersection targets, we identified that Sandwicensin could regulate molecular functions such as protein kinase activity and protein tyrosine kinase activity; biological processes such as protein phosphorylation, transferase activity, cellular lipid response, cell cycle and apoptosis; and cellular components such as γ-secretase complex. Meanwhile, the results of KEGG analysis revealed that the targets of Sandwicensin treatment for CRC were enriched in 15 signaling pathways (*p* < 0.05), as shown in Table S4 and Figure 5A and Figure 6A; the pathways with more than 10 mapped targets were pathways in cancer (hsa05200, number = 50), neuroactive ligand–receptor interactions (hsa04080, *n* = 11) and the cell cycle (hsa04110, *n* = 7). Notably, Sandwicensin treatment of CRC was strongly associated with cell cycle and apoptosis. This indicates that Sandwicensin may directly regulate the cycle, proliferation and apoptosis of tumor cells through cancer-related pathways.

## 3. Discussion

Cyclin D1 (CCND1), or G1/S-specific cyclin-D1, is a protein encoded by the human CCND1 gene [30,31]. The genes for cyclin D1 belong to a highly conserved cell cycle family. This family has cyclic changes in protein abundance throughout the cell cycle [32]. CDK, cyclin-dependent protein kinase, is a group of serine/threonine protein kinases [33]. CDK drives the cell cycle through chemotaxis on serine/threonine proteins and acts synergistically with cyclin, an important factor in cell cycle regulation, and CDK4 is an important isoform in the family [34]. A large number of studies have found that drugs can inhibit the growth of tumor cells by suppressing the expression of cyclins such as CCND1 and CDK4. Chai et al. found that interferon transmembrane protein 3 (IFITM3) knockdown reduces the expression of CCND1 and CDK4 and suppresses the growth of oral squamous cell carcinoma cells [35]. Wang et al. found that the knockdown of regeneration gene I-α (REGI-α) enhances the sensitivity to 5-fluorouracil of CRC cells via the cyclin D1/CDK4 pathway and BAX/BCL-2 pathways [36]. Ting et al. found that total ginsenoside (TGCG) induced cell cycle arrest in G0/G1 and G2/M phases through cMyc- and p53-mediated signaling pathways, and induced HT-29 apoptosis in CRC cells by regulating changes in protein levels of CCND1 and CDK4 [37]. The results of network pharmacology showed that Sandwicensin could regulate CCND1 and CDK4 to exert anti-CRC effects; notably, the results of KEGG enrichment analysis showed that Sandwicensin treatment of CRC was also closely linked to the cell cycle, suggesting that Sandwicensin exerts its anti-tumor effects mainly by regulating multiple cell cycle-related proteins. In addition, the mitogen-activated protein kinase (MAPK) and mammalian target of rapamycin (MTOR) pathway plays a potential role in cell proliferation and differentiation [38]. It has been shown that aberrant expression of anillin (ANLN) can regulate CRC cell proliferation through PI3K/AKT and MAPK pathways [39]. RBP-J promotes cell growth and metastasis by regulating the miR-182-5p-mediated Tiam1/Rac1/p38 MAPK axis in CRC [40]. Naringin inhibits CRC cell growth by repressing the PI3K/AKT/mTOR signaling pathway [41]. Our results show that Sandwicensin can also exert anti-tumor effects by regulating multiple cell proliferation-associated proteins.

## 4. Materials and Methods

### 4.1. General Information

Recording of 1D-NMR spectra was performed on a Bruker AV-600 spectrometer (Bruker, Billerica, MA, USA) with a single NMR probe at 300 MHz for ^1^H and 600 MHz for ^13^C in CDCl_3_. Column chromatographic silica gel (300 mesh) and TLC plates (GF254) were purchased from Qingdao Marine Chemical Inc. (Qingdao, China). The other solvents were purchased from Shanghai Titan Scientific Co., Ltd. (Shanghai, China).

### 4.2. Plant Material

*Pueraria lobata* (Willd.) Ohwi were purchased at Beiyao Pharmaceutical Group Co., Ltd., Jilin Province, China, in May 2021 and authenticated by Li-Li Weng, a professor at Changchun University of Traditional Chinese Medicine.

### 4.3. Cell Materials

DMEM high glucose medium (C11995500BT) and fetal bovine serum FBS (30044333) were from Gibco. PBS (SH30256), trypsin (SH30042), and penicillin–streptomycin dual antibody (SV30010) were all from Hyclone. Cell Counting Kit-8 kit (IV08-500) was from Invigentech. Propidium iodide (CF0031) was from Dingguo.

### 4.4. Extraction and Isolation

*Pueraria lobata* (10 kg) was extracted with 70% EtOH (34 L × 3 times, 7 days each time) and concentrated under reduced pressure to obtain 4.9 kg of crude ethanol extract. The crude ethanol extract of *Pueraria lobata* was dissolved according to a certain proportion (extract:distilled water = 1:1.2, V/V), and then liquid–liquid extraction was performed with dichloromethane, ethyl acetate and n-butanol in turn (aqueous phase:organic phase = 1:1, V/V), followed by concentration under reduced pressure to obtain the extract of each layer. Dichloromethane extracts were separated by silica gel column chromatography (CC) and gradient elution with petroleum ether–ethyl acetate (petroleum ether:ethyl acetate = 12:1→8:1→5:1→3:1, V/V) to obtain nine fractions (D-1→D-9).

Fr.D-2 (0.75 g) was further separated by a normal phase silica gel column (2.0 × 60 cm) with gradient elution with petroleum ether–dichloromethane and petroleum ether–ethyl acetate (petroleum ether:dichloromethane = 20:1→10:1→5:1; petroleum ether:ethyl acetate = 40:1, V/V) to afford a total of 16 fractions (D-2-1→D-2-16). Fr.D-2-9 (0.135 g) was further separated on a reversed-phase silica gel column (2.5 × 35 cm) with gradient elution with methanol–purified water and methanol–acetone (methanol:purified water = 9.3:0.7→9.8:0.2; methanol:acetone = 7:3, V/V) to afford compound **1** (51.1 mg).

Fr.D-4 (4.01 g) was further separated by a normal phase silica gel column (2.5 × 30 cm) and gradient elution with petroleum ether–dichloromethane (petroleum ether:dichloromethane = 5:1→2:1→0:1, V/V) to afford a total of four fractions (D-4-1→D-4-4). Fr.D-4-2 (0.969 g) was further separated by reversed-phase silica gel column (2.5 × 30 cm) and gradient elution with methanol–purified water (methanol:purified water = 8.2:1.8 →8.5:1.5→9.5:0.5, V/V) to give compound **2** (86.7 mg). Fr.D-4-3 (2.803 g) was further separated by reversed-phase silica gel column (2.5 × 45 cm) and gradient elution with methanol–purified water (methanol:purified water = 8:2→8.5:1.5→9.8:0.2, V/V) to afford compounds **3** and **4** (24.92 mg, 1.8699 g). Fr.D-4-4 (0.483 g) was further separated by reversed-phase silica gel column (2.5 × 45 cm) and gradient elution with methanol–purified water (methanol:purified water = 7:3→7.5:2.5→8:2, V/V) to give compound **5** (8.9 mg). Fr.D-4-4-8 (0.0082 g) was further separated by a normal phase silica column (1.2 × 20 cm) and eluted with petroleum ether–ethyl acetate (petroleum ether:ethyl acetate = 20:1, V/V) to give compound **6** (9.2 mg).

Fr.D-6 (2.04 g) was further separated by a normal phase silica gel column (2.5 × 30 cm) and gradient elution with petroleum ether–dichloromethane (petroleum ether:dichloromethane = 2:1→1:1, V/V) to give a total of nine fractions (D-6-1→D-6-9). Fr.D-6-3 (0.177 g) was separated on a reversed-phase silica gel column (2.5 × 45 cm) with gradient elution with methanol–purified water (methanol:purified water = 4.5:5.5→6.5:3.5→7:3→8:2→9:1, V/V) to afford compounds **7** and **8** (30.9 mg,3 mg). Fr.D-6-7 (0.205 g) was further separated on a reversed-phase silica gel column (2.5 × 45 cm) with gradient elution with methanol–purified water (methanol:purified water = 5.2:4.8→6:4→7.3:2.7→8.5:2.5, V/V) to give compound **9** (24.5 mg).

Fr.D-7 (3.91 g) was further separated by a normal phase silica gel column (2.5 × 30 cm) and gradient elution with petroleum ether–ethyl acetate (petroleum ether:ethyl acetate = 8:1, V/V) to afford a total of four fractions (D-7-1→D-7-4). Fr.D-7-2 (1.4 g) was further separated by reversed-phase silica gel column (3.5 × 45 cm) and gradient elution with methanol–purified water (methanol:purified water = 3.3:6.7→4:6→5.5:4.5→6.7:2.3, V/V) to give compounds **10**–**12** (4.7 mg, 3.7 mg, 27.8 mg).

Fr.D-8 (7.13 g) was further separated by a normal phase silica gel column (5 × 45 cm) and gradient elution with petroleum ether–ethyl acetate (petroleum ether:ethyl acetate = 7:1, V/V) to give a total of five fractions (D-8-1→D-8-5). Fr.D-8-2 (0.197 g) was further separated by reverse silica gel column (2.5 × 45 cm) and gradient elution with methanol–purified water (methanol:purified water = 5.2:4.8→5.5:4.5→6.5:3.5→8:2, V/V) to give compound **13** (9.47 mg). Fr.D-8-5-14 (0.016 g) was further separated by preparing thin layer plates (20 × 20 cm) with dichloromethane:methanol = 20:1 as developing solvent to give compound **14** (12 mg).

### 4.5. Preparative Thin Layer Plates

Test article solution was spotted on preparative TLC (1 mm, 20×20 cm), with dichloromethane:methanol = 20:1 as developing solvent; the TLC plate was taken out after developing, the solvent was evaporated to dryness, the plated was observed under an ultraviolet lamp and 10% sulfuric acid was used to develop color.

### 4.6. Characterization

NMR spectroscopy was performed by Jilin University.

### 4.7. Cell Culture

SW480 and SW620 cells were cultured in DMEM supplemented with 10% fetal bovine serum (FBS) and 1% penicillin–streptomycin at 37 °C in a humidified incubator (5% CO_2_ and 95% air). The medium was changed every other day, and the cells were subcultured when they reached 80% confluence.

### 4.8. Cytotoxicity Assessment

The cells were inoculated in 96-well plates at a density of 5 × 10^3^ per well, and after the cells were fully attached to the wall, they were incubated with different concentrations of drugs for 48 h. After incubation, 20 μL of CCK-8 solution was added to each well for 30 min, and the absorbance (A) value was measured at 450 nm to calculate the cell survival rate.

Cell survival rate = A (drug administration group − blank group)/A (control group − blank group) × 100% (1)

### 4.9. Network Pharmacology Technology

“Sandwicensin” was entered in the TCMSP database (http://lsp.nwu.edu.cn/tcmsp.php, accessed on 20 September 2022) to collect the targets of this component; the structure of Sandwicensin was drawn in the Swiss Target Prediction database (http://www.Swiss Target Prediction.ch, accessed on 20 September 2022), and the predicted targets corresponding to this component were collected [42,43].

“Colorectal cancer” and “colorectal carcinoma” for all targets of the disease were collected in the OMIM database (https://omim.org/, accessed on 20 September 2022), GeneCards database (https://www.genecards.org/), Disgenet database (https://www.disgenet.org/, accessed on 20 September 2022), TTD database and Drugbank database and then logged in UniProt (http://www.uniprot.org/, accessed on 20 September 2022) database, and the gene names and Uniprot IDs of the targets were corrected [44,45,46,47,48]. The components and disease targets were collated, de-weighted and mapped, and VENE maps were drawn.

The targets after component and disease mapping are the key targets of Sandwicensin for the treatment of CRC. After the component and disease target mapping, Cytoscape 3.2.1 software (http://www.cytoscape.org, accessed on 20 September 2022) was used to construct the “component–target” relationship network. This network can be used to analyze the mechanism of action of Sandwicensin in the treatment of CRC [49].

Using the “Multiple Proteins” function of the STRING database (https://string-db.org, accessed on 20 September 2022) [50], we selected “Organism” as Homo Sapiens, imported the key targets of Sandwicensin for CRC into the database with a confidence score >0.95, and hid the free nodes. The key targets of Sandwicensin for CRC were imported into this database, the confidence score > 0.95 was searched, and the free nodes were hidden to draw protein–protein interaction (PPI) networks. Cytoscape 3.2.1 software was used to perform network analysis and rank the key targets according to the degree of freedom value.

The key targets of Sandwicensin for CRC were entered into the Math Escape online analysis tool (https://david.ncifcrf.gov/, accessed on 20 September 2022) for GO function enrichment analysis and KEGG pathway enrichment analysis. The information was collated separately, and bubble maps were plotted in the Microbioxin database.

### 4.10. Statistical Analysis

The experimental data were entered into Graphpad Prism 7 and analyzed by *t*-test and analysis of variance (ANOVA). Statistical significance was determined based on *p* values (* *p* < 0.05, ** *p* < 0.01, *** *p* < 0.001). All results are expressed as mean ± SD, *n* = 3.

## 5. Conclusions

In the present study, 14 compounds were isolated and purified from the dichloromethane extract of *Pueraria lobata*, including 7 compounds that were isolated from this plant for the first time. In addition, compounds **5**, **6**, **9**, **11** and **12** showed good anti-tumor activity, with compound **5** having the best biological activity. This indicates that compound **5** is the main bioactive component of the anti-tumor activity of *Pueraria lobata*. Moreover, we used network pharmacology to mine and analyze the targets and signaling pathways of Sandwicensin for the treatment of CRC, and the results showed that Sandwicensin exerts its anti-tumor effects mainly through regulating several cell cycle-related proteins (CCND1, CDK4, etc.) and several proliferation-related proteins (MAPK3, MTOR, etc.).

## Data Availability

All data are available in this publication and in the Supplementary Materials.

## Supplementary Materials

The following supporting information can be downloaded at: https://www.mdpi.com/article/10.3390/molecules27217253/s1, Table S1: Potential core targets; Table S2: Potential core target interactions; Table S3: GO annotation enrichment analysis; Table S4: KEGG pathway enrichment analysis.

## Sample Availability

Samples of the compounds are not available from the authors.

## References

[1] Chang K.F., Chang J.T., Huang X.F., Lin Y.L., Liao K.W., Huang C.W., Tsai N.M. (2020). Antitumor effects of N-butylidenephthalide encapsulated in lipopolyplexs in colorectal cancer cells. Molecules.

[2] Uyemura S.A., Stopper H., Martin F.L., Kannen V. (2017). A perspective discussion on rising pesticide levels and colon cancer burden in brazil. Front. Public Health.

[3] Gao Y., Xiao X., Zhang C., Yu W., Guo W., Zhang Z., Li Z., Feng X., Hao J., Zhang K. (2017). Melatonin synergizes the chemotherapeutic effect of 5-fluorouracil in colon cancer by suppressing PI3K/AKT and NF-kappaB/iNOS signaling pathways. J. Pineal Res..

[4] Ma S., Lei Y., Zhang L., Wang J. (2018). Research on the inhibiting effect of tanshinone IIA on colon cancer cell growth via COX-2-Wnt/beta-catenin signaling pathway. J. BUON.

[5] Song Q., Sun X., Guo H., Yu Q. (2017). Concomitant inhibition of receptor tyrosine kinases and downstream AKT synergistically inhibited growth of KRAS/BRAF mutant colorectal cancer cells. Oncotarget.

[6] Wang Z., Qi F., Cui Y., Zhao L., Sun X., Tang W., Cai P. (2018). An update on Chinese herbal medicines as adjuvant treatment of anticancer therapeutics. Biosci. Trends.

[7] Zhang L. (2019). Pharmacokinetics and drug delivery systems for puerarin, a bioactive flavone from traditional Chinese medicine. Drug Deliv..

[8] Shi W., Yuan R., Chen X., Xin Q., Wang Y., Shang X., Cong W., Chen K. (2019). Puerarin eeduces blood pressure in spontaneously hypertensive rats by targeting eNOS. Am. J. Chin. Med..

[9] Kim Y.J., Kim H.J., Ok H.M., Jeong H.Y., Lee W.J., Weaver C., Kwon O. (2018). Effect and interactions of Pueraria-Rehmannia and aerobic exercise on metabolic inflexibility and insulin resistance in ovariectomized rats fed with a high-fat diet. J. Funct. Foods.

[10] Jearapong N., Chatuphonprasert W., Jarukamjorn K. (2014). Miroestrol, a phytoestrogen from Pueraria mirifica, improves the antioxidation state in the livers and uteri of beta-naphthoflavone-treated mice. J. Nat. Med..

[11] Shukla R., Banerjee S., Tripathi Y.B. (2018). *Pueraria tuberosa* extract inhibits iNOS and IL-6 through suppression of PKC-alpha and NF-kB pathway in diabetes-induced nephropathy. J. Pharm. Pharmacol..

[12] Satpathy S., Patra A., Ahirwar B., Delwar Hussain M. (2018). Antioxidant and anticancer activities of green synthesized silver nanoparticles using aqueous extract of tubers of *Pueraria tuberosa*. Artif. Cells Nanomed. Biotechnol..

[13] Zhang X.L., Wang B.B., Mo J.S. (2018). Puerarin 6”-O-xyloside possesses significant antitumor activities on colon cancer through inducing apoptosis. Oncol. Lett..

[14] Satpathy S., Patra A., Hussain M.D., Kazi M., Aldughaim M.S., Ahirwar B. (2021). A fraction of *Pueraria tuberosa* extract, rich in antioxidant compounds, alleviates ovariectomized-induced osteoporosis in rats and inhibits growth of breast and ovarian cancer cells. PLoS ONE.

[15] Ahn E.K., Oh J.S. (2013). Lupenone isolated from Adenophora triphylla var. japonica extract inhibits adipogenic differentiation through the downregulation of PPARgamma in 3T3-L1 cells. Phytother. Res..

[16] Huang H.M., Ho C.Y., Chang G.R., Shia W.Y., Lai C.H., Chao C.H., Wang C.M. (2021). HPLC/ESI-MS and NMR analysis of chemical constitutes in bioactive extract from the root nodule of vaccinium emarginatum. Pharmaceuticals.

[17] Zhou S. (2013). Preliminary Study on Chemical Composition and the Activity of Dracocephalum Heterophyllum. Ph.D. Thesis.

[18] Ododo M.M., Choudhury M.K., Dekebo A.H. (2016). Structure elucidation of beta-sitosterol with antibacterial activity from the root bark of Malva parviflora. SpringerPlus.

[19] McKee T.C., Bokesch H.R., McCormick J.L., Rashid M.A., Spielvogel D., Gustafson K.R., Alavanja M.M., Cardelline J.H., Boyd M.R. (1997). Isolation and characterization of new anti-HIV and cytotoxic leads from plants, marine, and microbial organisms. J. Nat. Prod..

[20] Mitscher L.A., Gollapudi S.R., Gerlach D.C., Drake S.D., Eduardo A.V., Ward J.A. (1988). Erycristin, a new antimicrobial petrocarpan from Erythrina crista-galli. Phytochemistry.

[21] He J., Fan P., Feng S., Shao P., Sun P. (2018). Isolation and purification of two isoflavones from hericium erinaceum mycelium by high-speed counter-current chromatography. Molecules.

[22] Ryu J., Son D., Kang J., Kim H.S., Kim B.K., Lee S. (2004). A benzenoid from the stem of Acanthopanax senticosus. Arch. Pharmacal Res..

[23] Jin Q., Jia-hong Z., Lu C., Ji-jun C., Bo-yang Y., Sheng-xiang Q. (2006). Study on chemical constituents in tuber of harpagophytum procumbens D.C. Chin. Pharm. J..

[24] Hasan N., Osman H., Mohamad S., Chong W.K., Awang K., Zahariluddin A.S. (2012). The chemical components of sesbania grandiflora root and their antituberculosis activity. Pharmaceuticals.

[25] Ghanadian M., Ali Z., Khan I.A., Balachandran P., Nikahd M., Aghaei M., Mirzaei M., Sajjadi S.E. (2020). A new sesquiterpenoid from the shoots of Iranian Daphne mucronata Royle with selective inhibition of STAT3 and Smad3/4 cancer-related signaling pathways. Daru.

[26] Miyase T., Ueno A., Noro T., Fukushima S. (1980). Studies on the Constituentes of Lespedeza cyrtobotrya MIQ. I. The structures of a new chalcone and two new isoflav-3-ens. Chem. Pharm. Bull..

[27] Dewick P.M., Ingham J.L. (1980). Isopterofuran, a new 2-arylbenzofuran phytoalexin from Coronilla emerus. Phytochemistry.

[28] Luk K.C., Stern L., Weigele M., O’Brien R.A., Spirt N. (1983). Isolation and identification of “diazepam-like” compounds from bovine urine. J. Nat. Prod..

[29] Kumar A.R., Selvaraj S., Jayaprakash K.S., Gunasekaran S., Kumaresan S., Devanathan J., Selvam K.A., Ramadass L., Mani M., Rajkumar P. (2021). Multi-spectroscopic (FT-IR, FT-Raman, 1H NMR and 13C NMR) investigations on syringaldehyde. J. Mol. Struct..

[30] Chen S., Li L. (2022). Degradation strategy of cyclin D1 in cancer cells and the potential clinical application. Front. Oncol..

[31] Montalto F.I., De Amicis F. (2020). Cyclin D1 in Cancer: A molecular connection for cell cycle control, adhesion and invasion in tumor and stroma. Cells.

[32] Gonzalez-Ruiz L., Gonzalez-Moles M.A., Gonzalez-Ruiz I., Ruiz-Avila I., Ramos-Garcia P. (2021). Prognostic and clinicopathological significance of CCND1/Cyclin D1 upregulation in melanomas: A systematic review and comprehensive meta-analysis. Cancers.

[33] Goel B., Tripathi N., Bhardwaj N., Jain S.K. (2020). Small molecule CDK inhibitors for the therapeutic management of cancer. Curr. Top. Med. Chem..

[34] Nardone V., Barbarino M., Angrisani A., Correale P., Pastina P., Cappabianca S., Reginelli A., Mutti L., Miracco C., Giannicola R. (2021). CDK4, CDK6/cyclin-D1 complex inhibition and radiotherapy for cancer control: A Role for Autophagy. Int. J. Mol. Sci..

[35] Gan C.P., Sam K.K., Yee P.S., Zainal N.S., Lee B.K.B., Abdul Rahman Z.A., Patel V., Tan A.C., Zain R.B., Cheong S.C. (2019). IFITM3 knockdown reduces the expression of CCND1 and CDK4 and suppresses the growth of oral squamous cell carcinoma cells. Cell. Oncol..

[36] Wang Y., Liu X., Liu J., Zhang T. (2019). Knockdown of REG ialpha enhances the sensitivity to 5-fluorouracil of colorectal cancer cells via Cyclin D1/CDK4 pathway and BAX/BCL-2 pathways. Cancer Biother. Radiopharm..

[37] Li T., Sun W., Dong X., Yu W., Cai J., Yuan Q., Shan L., Efferth T. (2018). Total ginsenosides of chinese ginseng induces cell cycle arrest and apoptosis in colorectal carcinoma HT-29 cells. Oncol. Lett..

[38] Wu Q., Liu T.Y., Hu B.C., Li X., Wu Y.T., Sun X.T., Jiang X.W., Wang S., Qin X.C., Ding H.W. (2021). CK-3, A novel methsulfonyl pyridine derivative, suppresses hepatocellular carcinoma proliferation and invasion by blocking the PI3K/AKT/mTOR and MAPK/ERK pathways. Front. Oncol..

[39] Liu Y., Cao P., Cao F., Wang S., He Y., Xu Y., Wang Y. (2022). ANLN, Regulated by SP2, promotes colorectal carcinoma cell proliferation via PI3K/AKT and MAPK signaling pathway. J. Investig. Surg..

[40] Li F., Zhou Y.D., Liu J., Cai J.D., Liao Z.M., Chen G.Q. (2021). RBP-J promotes cell growth and metastasis through regulating miR-182-5p-mediated Tiam1/Rac1/p38 MAPK axis in colorectal cancer. Cell Signal.

[41] Cheng H., Jiang X., Zhang Q., Ma J., Cheng R., Yong H., Shi H., Zhou X., Ge L., Gao G. (2020). Naringin inhibits colorectal cancer cell growth by repressing the PI3K/AKT/mTOR signaling pathway. Exp. Ther. Med..

[42] Gfeller D., Grosdidier A., Wirth M., Daina A., Michielin O., Zoete V. (2014). SwissTargetPrediction: A web server for target prediction of bioactive small molecules. Nucleic Acids Res..

[43] Ru J., Li P., Wang J., Zhou W., Li B., Huang C., Li P., Guo Z., Tao W., Yang Y. (2014). TCMSP: A database of systems pharmacology for drug discovery from herbal medicines. J. Cheminform..

[44] Amberger J.S., Hamosh A. (2017). Searching Online Mendelian Inheritance in Man (OMIM): A Knowledgebase of human genes and genetic phenotypes. Curr. Protoc. Bioinform..

[45] Pinero J., Ramirez-Anguita J.M., Sauch-Pitarch J., Ronzano F., Centeno E., Sanz F., Furlong L.I. (2020). The DisGeNET knowledge platform for disease genomics: 2019 update. Nucleic Acids Res..

[46] Stelzer G., Rosen N., Plaschkes I., Zimmerman S., Twik M., Fishilevich S., Stein T.I., Nudel R., Lieder I., Mazor Y. (2016). The genecards suite: From gene data mining to disease genome sequence analyses. Curr. Protoc. Bioinform..

[47] UniProt C. (2021). UniProt: The universal protein knowledgebase in 2021. Nucleic Acids Res..

[48] Wishart D.S., Feunang Y.D., Guo A.C., Lo E.J., Marcu A., Grant J.R., Sajed T., Johnson D., Li C., Sayeeda Z. (2018). DrugBank 5.0: A major update to the drugBank database for 2018. Nucleic Acids Res..

[49] Shannon P., Markiel A., Ozier O., Baliga N.S., Wang J.T., Ramage D., Amin N., Schwikowski B., Ideker T. (2003). Cytoscape: A software environment for integrated models of biomolecular interaction networks. Genome Res..

[50] Szklarczyk D., Gable A.L., Nastou K.C., Lyon D., Kirsch R., Pyysalo S., Doncheva N.T., Legeay M., Fang T., Bork P. (2021). The STRING database in 2021: Customizable protein-protein networks, and functional characterization of user-uploaded gene/measurement sets. Nucleic Acids Res..

