# Supplementary material for: Studies on Chemical Composition of Pueraria lobata and Its Anti-Tumor Mechanism
Source: Molecules. 2022 Oct 26;27(21):7253. doi: 10.3390/molecules27217253 (PMC9657109; doi:10.3390/molecules27217253)
Supplement: Supplementary file 1 [file molecules-27-07253-s001.zip › molecules-1982753-supplementary.pdf]

Studies on Chemical Composition of *Pueraria lobata* and Its Anti-tumor Mechanism

Table S1. Potential core targets.

Number	Target name	Degree	Number	Target name	Degree
1	MAPK3	38	48	PIM2	7
2	MTOR	38	49	PLAT	7
3	HSP90AA1	37	50	PSENEN	7
4	CCND1	36	51	CRHR1	6
5	MAPK1	33	52	ERN1	6
6	PIK3CA	33	53	LDLR	6
7	ESR1	32	53	PDE10A	6
8	GSK3B	25	55	ROCK1	6
9	JAK2	25	56	ADORA2A	5
10	MCL1	25	57	ADORA2B	5
11	CDK4	24	58	GABRA1	5
12	EP300	22	59	HSD17B2	5
13	ABL1	20	60	HSD17B3	5
14	AR	20	61	PDE5A	5
15	RAF1	18	62	S1PR1	5
16	CCND2	17	63	GRM4	4
17	MET	17	64	PDE2A	4
18	CHEK1	16	65	PDE7A	4
19	RPS6KB1	16	66	PIM1	4
20	CCND3	15	67	PLK4	4
21	CDK5	14	68	VCP	4
22	PLK1	14	69	ADAMTS5	3
23	AURKA	13	70	CPT1A	3
24	LCK	13	71	DRD4	3
25	PSEN1	12	72	GABRA6	3
26	WEE1	12	73	MMP8	3
27	BRAF	11	74	PFKFB3	3
28	CYP19A1	11	75	SCD	3
29	FLT3	11	76	TGFBR1	3
30	PSEN2	11	77	TRPV1	3
31	ESR2	10	78	ANPEP	2
32	PRKCG	10	79	CASR	2
33	PTPN1	10	80	FNTA	2
34	RET	10	81	MAOA	2
35	RPS6KA1	10	82	MIF	2
36	BAD	9	83	TSPO	2
37	IDH1	9	84	TYR	2

38	MMP1	9	85	CCR3	1
39	MMP3	9	86	CMA1	1
40	PIK3CG	9	87	CTSS	1
41	PLAU	9	88	FNTB	1
42	ROCK2	9	89	FTO	1
43	APH1A	8	90	MERTK	1
44	EPHB4	8	91	RIPK2	1
45	NCSTN	8	92	TBXA2R	1
46	APH1B	7	93	TNNI3K	1
47	PDE4A	7	—	—	—

Table S2. Potential core target interactions.

Node1	Node2	Coexp ression	Experimentally determined interaction	Database annotated	Automated textmining	Combined score
APH1A	NCSTN	0.088	0.985	0.9	0.995	0.999
APH1A	PSEN1	0.066	0.982	0.9	0.993	0.999
APH1A	PSEN2	0.066	0.786	0.8	0.984	0.999
APH1A	PSENEN	0.088	0.984	0.9	0.995	0.999
AR	HSP90AA1	0	0.884	0.9	0.989	0.999
AURKA	PLK1	0.946	0.786	0.9	0.956	0.999
CCND1	CDK4	0.08	0.998	0.9	0.993	0.999
CCND1	ESR1	0	0.867	0.9	0.987	0.999
CCND2	CDK4	0.066	0.948	0.9	0.993	0.999
CCND3	CDK4	0.066	0.99	0.9	0.993	0.999
CDK4	HSP90AA1	0.062	0.889	0	0.991	0.999
EP300	ESR1	0	0.929	0.9	0.988	0.999
ESR1	HSP90AA1	0	0.887	0.6	0.989	0.999
FNTA	FNTB	0.087	0.996	0.9	0.937	0.999
HSP90AA1	RAF1	0	0.942	0	0.988	0.999
MTOR	RPS6KB1	0.062	0.992	0.9	0.951	0.999
NCSTN	PSEN1	0.083	0.989	0.9	0.994	0.999
NCSTN	PSEN2	0.063	0.839	0.8	0.994	0.999
NCSTN	PSENEN	0.062	0.987	0.9	0.995	0.999
PSEN1	PSENEN	0.062	0.982	0.9	0.993	0.999
PSEN2	PSENEN	0.062	0.908	0.8	0.976	0.999
APH1B	NCSTN	0.065	0.646	0.9	0.965	0.998
AR	CCND1	0.061	0.839	0.9	0.921	0.998

MAPK1	RPS6KA1	0.062	0.98	0.9	0.972	0.998
APH1B	PSENEN	0.088	0.519	0.9	0.947	0.997
AR	EP300	0	0.881	0.9	0.793	0.997
JAK2	PTPN1	0.062	0.856	0.9	0.858	0.997
APH1B	PSEN1	0.066	0.646	0.9	0.895	0.996
CHEK1	HSP90AA1	0.062	0.655	0	0.988	0.996
CCND1	EP300	0.062	0.835	0.9	0.754	0.995
BRAF	RAF1	0	0.934	0.9	0.982	0.993
CCND1	GSK3B	0	0.6	0.9	0.845	0.993
GSK3B	PSEN1	0.089	0.79	0.8	0.832	0.992
BAD	RAF1	0	0.876	0.9	0.259	0.99
HSP90AA1	MAPK3	0.054	0.299	0.9	0.866	0.99
JAK2	PIK3CG	0.106	0.104	0.9	0.889	0.99
MAPK1	RAF1	0.066	0.846	0.9	0.738	0.989
HSP90AA1	TGFBR1	0	0.715	0	0.96	0.988
MAPK1	MAPK3	0	0.887	0.9	0.923	0.988
BRAF	MAPK1	0.066	0.835	0.9	0.585	0.987
ESR1	ESR2	0	0.873	0.9	0.991	0.987
APH1B	PSEN2	0.066	0.661	0.8	0.808	0.986
MAPK3	RAF1	0.066	0.798	0.9	0.836	0.986
PIK3CA	RPS6KB1	0.065	0.354	0.9	0.809	0.986
ESR1	MAPK1	0	0.699	0.9	0.532	0.984
BRAF	MAPK3	0.066	0.757	0.9	0.702	0.982
ESR1	PIK3CA	0	0.496	0.9	0.685	0.982
MAPK3	RPS6KA1	0.062	0.759	0.9	0.813	0.982
HSD17B2	HSD17B3	0	0	0.8	0.912	0.981
MET	PTPN1	0.062	0.731	0.9	0.334	0.981
HSP90AA1	MAPK1	0.062	0.365	0.9	0.705	0.98
PLK1	WEE1	0.196	0.606	0.9	0.9	0.979
FLT3	HSP90AA1	0	0.306	0	0.97	0.978
AURKA	GSK3B	0	0.689	0.9	0.574	0.977
LCK	MAPK1	0	0.713	0.9	0.418	0.977
HSP90AA1	RPS6KB1	0.076	0.247	0.9	0.7	0.976

HSP90A A1	MTOR	0	0.092	0.9	0.763	0.976
MET	PIK3CA	0.063	0.104	0.9	0.753	0.976
LCK	PIK3CA	0.062	0.699	0.9	0.222	0.975
ABL1	HSP90A A1	0	0.829	0	0.856	0.974
ESR1	MAPK3	0	0.264	0.9	0.683	0.974
PIK3CA	RET	0	0.097	0.9	0.71	0.971
ROCK1	ROCK2	0.084	0.684	0.9	0.898	0.969
JAK2	PIK3CA	0.09	0.104	0.9	0.645	0.967
AR	GSK3B	0	0.482	0.9	0.405	0.966
CHEK1	WEE1	0.131	0.391	0.9	0.894	0.965
CYP19A1	HSD17B 2	0	0.072	0.9	0.655	0.965
CCND1	CDK5	0.052	0.401	0	0.941	0.964
CCND1	JAK2	0	0	0.9	0.655	0.964
ESR2	HSP90A A1	0	0.285	0.6	0.886	0.964
CYP19A1	HSD17B 3	0.052	0	0.9	0.65	0.963
ABL1	PTPN1	0.062	0.426	0.9	0.308	0.957
HSP90A A1	ROCK1	0.062	0.161	0.9	0.518	0.957
MTOR	PIK3CA	0.062	0.347	0.9	0.782	0.957
HSP90A A1	MCL1	0	0.128	0.9	0.536	0.956
MAPK3	RPS6KB 1	0.062	0.309	0.9	0.78	0.956
CDK5	GSK3B	0.062	0.47	0.9	0.791	0.955
HSP90A A1	VCP	0.157	0.453	0.6	0.787	0.955
GABRA1	GABRA 6	0.476	0.151	0.9	0.778	0.953
MMP1	MMP3	0.529	0	0.9	0.912	0.953
AR	PIK3CA	0	0.061	0.9	0.52	0.95
MAPK1	RPS6KB 1	0.062	0.309	0.9	0.594	0.95

Table S3. GO annotation enrichment analysis.

GO	Category	Description	Count
GO:0004672	GO Molecular Functions	protein kinase activity	37

GO:0006468	GO Biological Processes	protein phosphorylation	36
GO:0051347	GO Biological Processes	positive regulation of transferase activity	25
GO:0071396	GO Biological Processes	cellular response to lipid	23
GO:1901699	GO Biological Processes	cellular response to nitrogen compound	24
GO:0048732	GO Biological Processes	gland development	18
GO:0030162	GO Biological Processes	regulation of proteolysis	22
GO:0009725	GO Biological Processes	response to hormone	22
GO:0070765	GO Cellular Components	gamma-secretase complex	6
GO:0018105	GO Biological Processes	peptidyl-serine phosphorylation	13
GO:0031331	GO Biological Processes	positive regulation of cellular catabolic process	17
GO:0032103	GO Biological Processes	positive regulation of response to external stimulus	17
GO:0010506	GO Biological Processes	regulation of autophagy	15
GO:0044772	GO Biological Processes	mitotic cell cycle phase transition	11
GO:0051098	GO Biological Processes	regulation of binding	14
GO:2001233	GO Biological Processes	regulation of apoptotic signaling pathway	14
GO:0080135	GO Biological Processes	regulation of cellular response to stress	18
GO:0004713	GO Molecular Functions	protein tyrosine kinase activity	10
GO:0001775	GO Biological Processes	cell activation	17
GO:0007610	GO Biological Processes	behavior	16

Table S4. KEGG pathway enrichment analysis.

ID	**Description**	**Count**
hsa05200	Pathways in cancer	30
hsa04080	Neuroactive ligand–receptor interaction	11
hsa04110	Cell cycle	10
hsa04330	Notch signaling pathway	7
hsa05032	Morphine addiction	7
hsa04931	Insulin resistance	7
hsa05202	Transcriptional misregulation in cancer	6
hsa05415	Diabetic cardiomyopathy	6
hsa04020	Calcium signaling pathway	6
hsa04723	Retrograde endocannabinoid signaling	5
hsa04728	Dopaminergic synapse	4
hsa04936	Alcoholic liver disease	4
hsa00350	Tyrosine metabolism	3

hsa04913	Ovarian steroidogenesis	3
hsa04925	Aldosterone synthesis and secretion	3

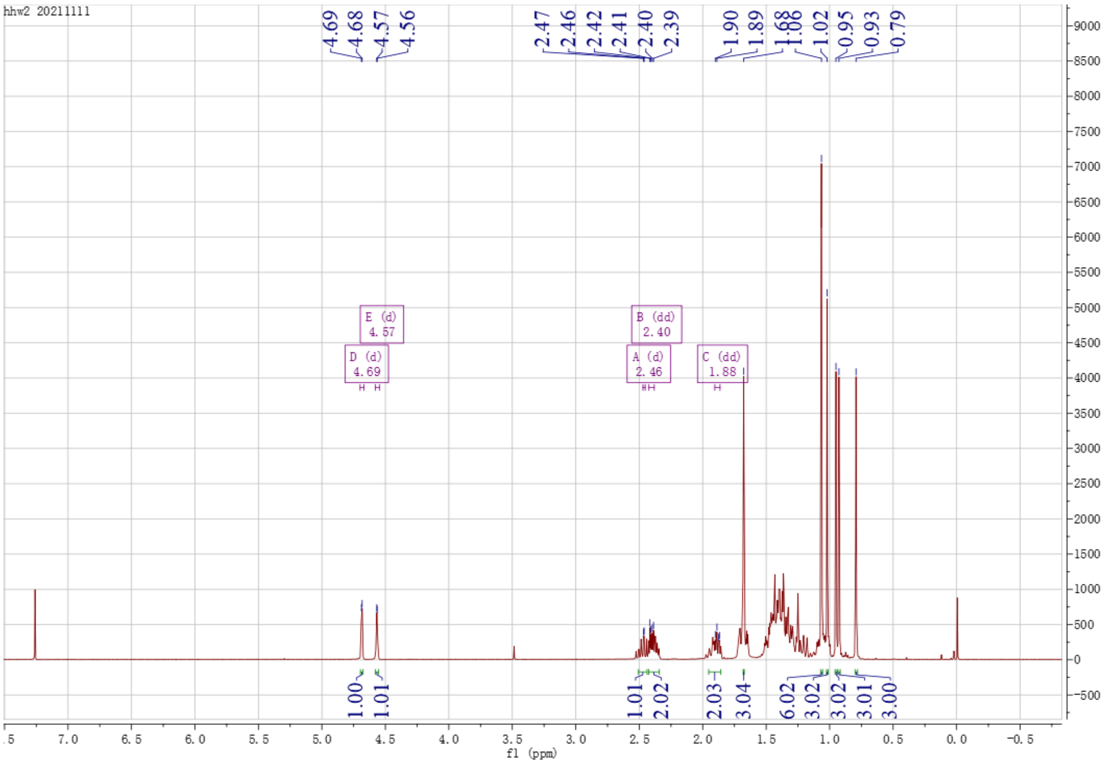

Compound 1 ^{1}H-NMR

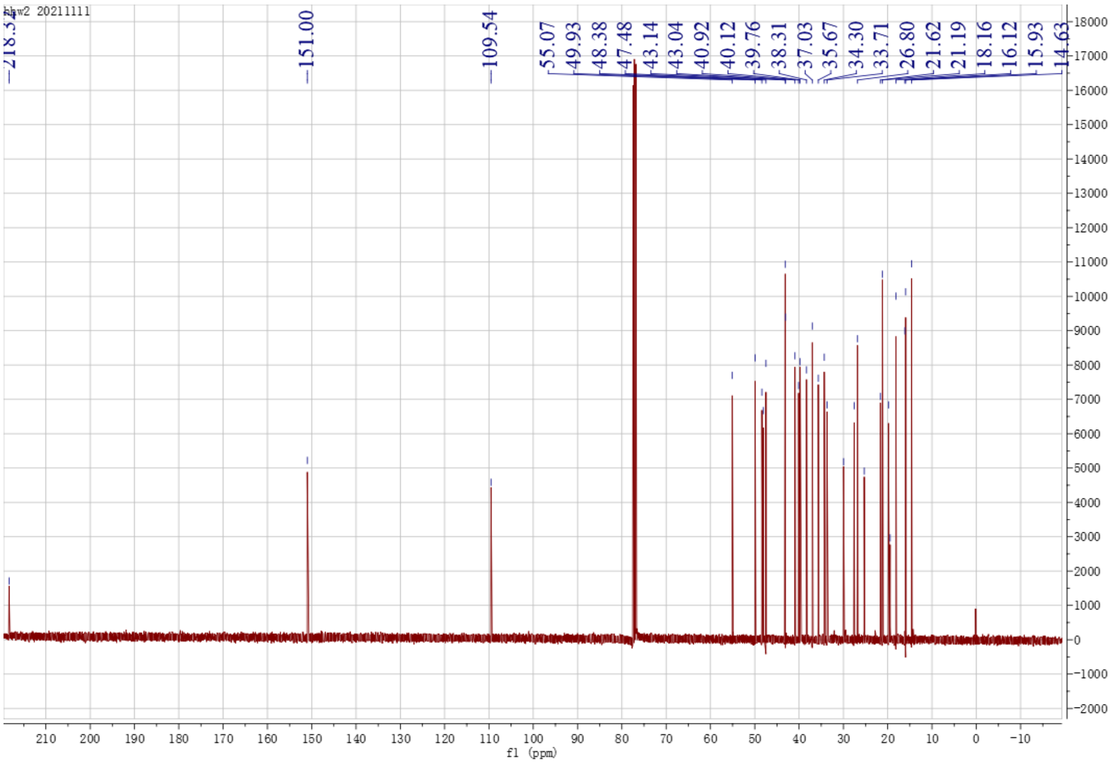

Compound 1 ^{13}C-NMR

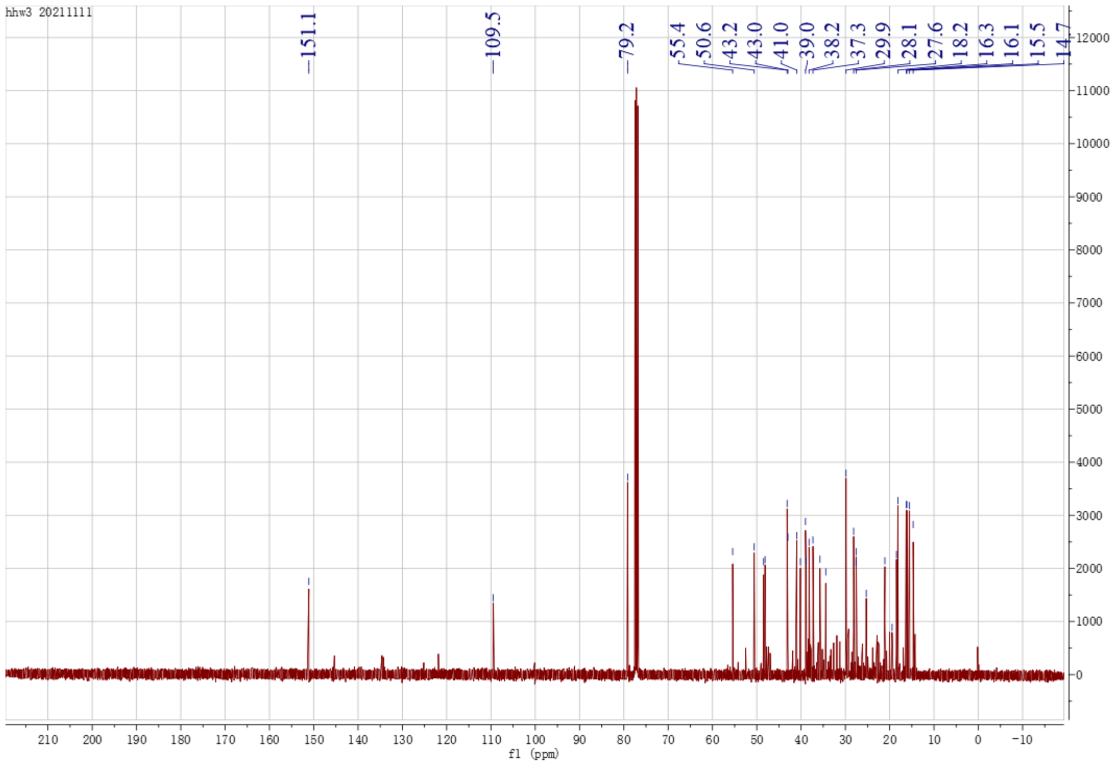

Compound 2 ^{13}C-NMR

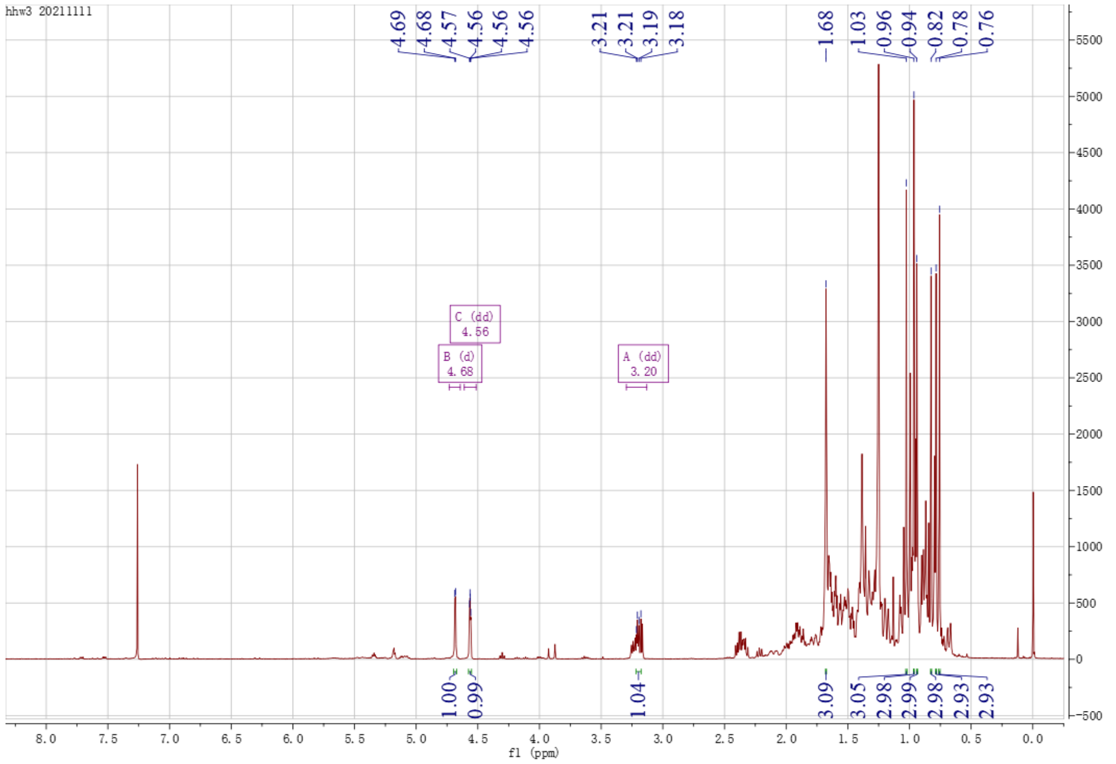

Compound 2 ^{1}H-NMR

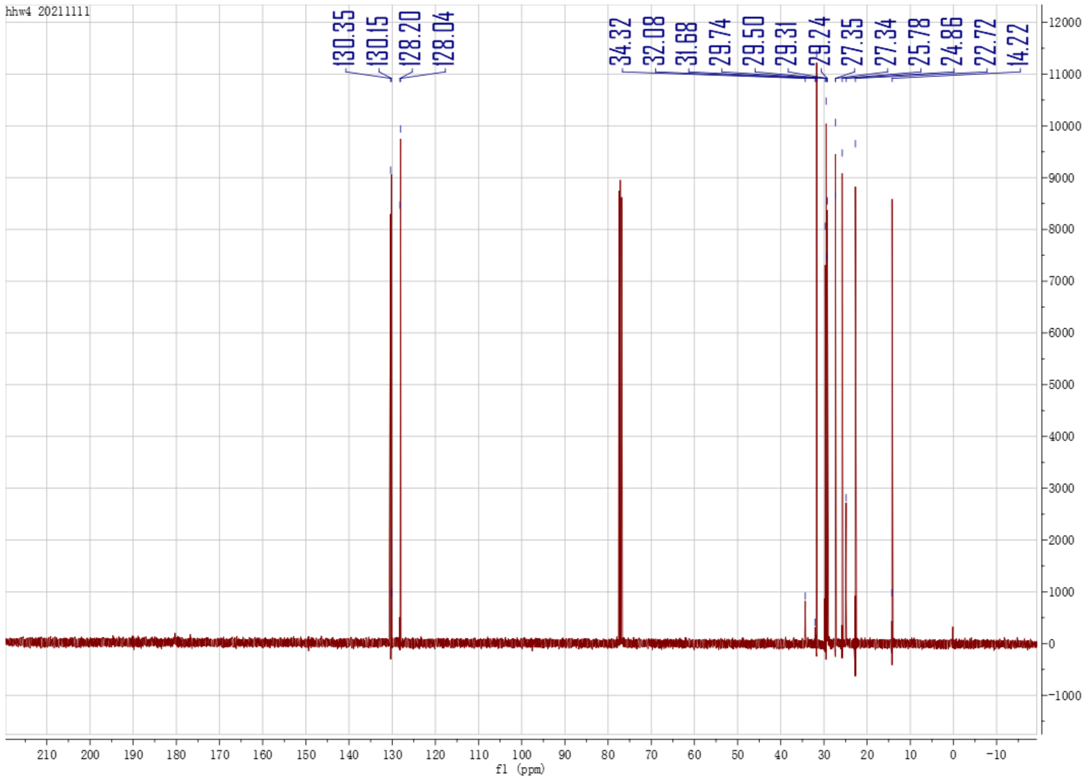

Compound 3 ^{13}C-NMR

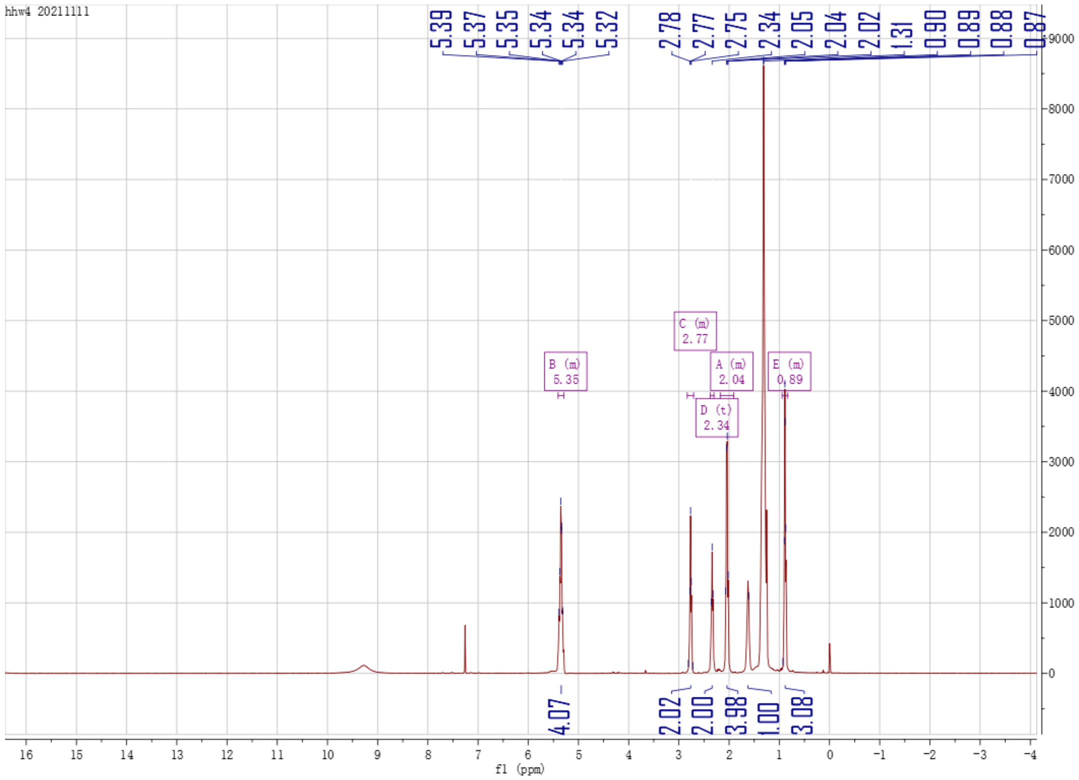

Compound 3 ^{1}H-NMR

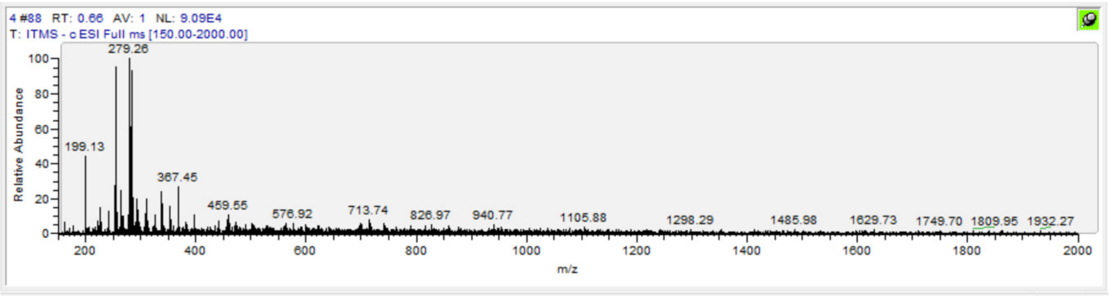

Compound 3 M/Z

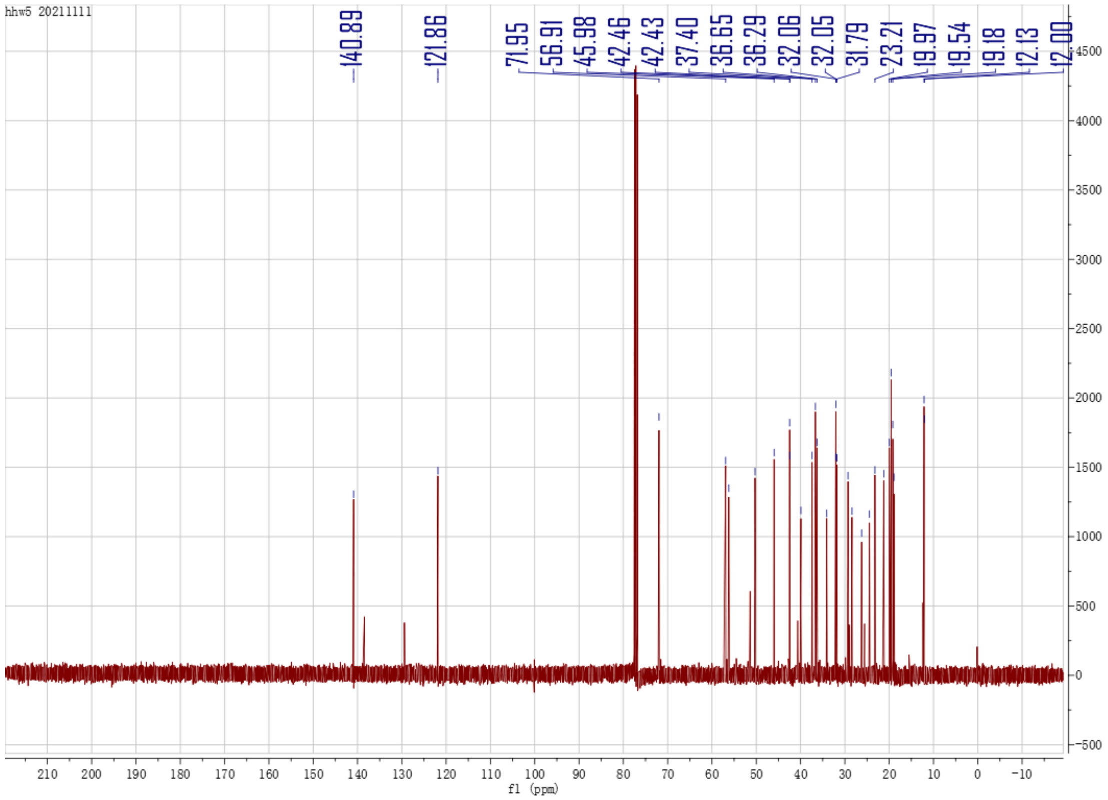

Compound 4 ^{13}C-NMR

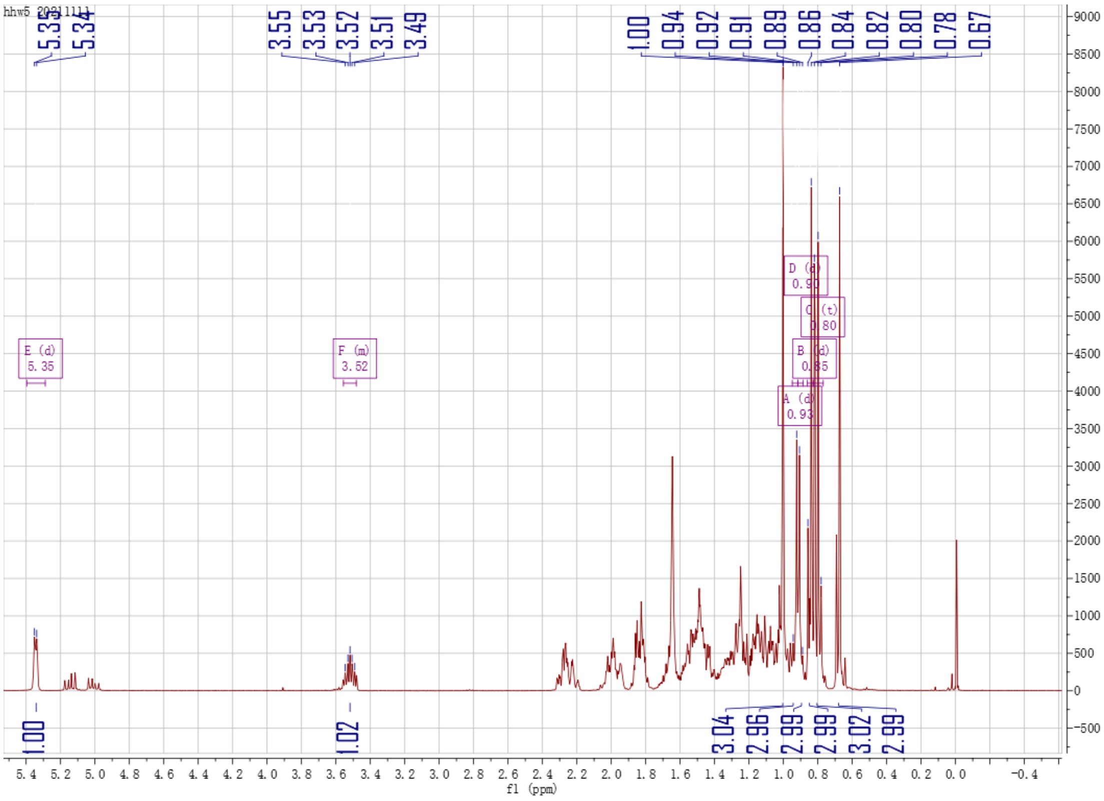

Compound 4 ^{1}H-NMR

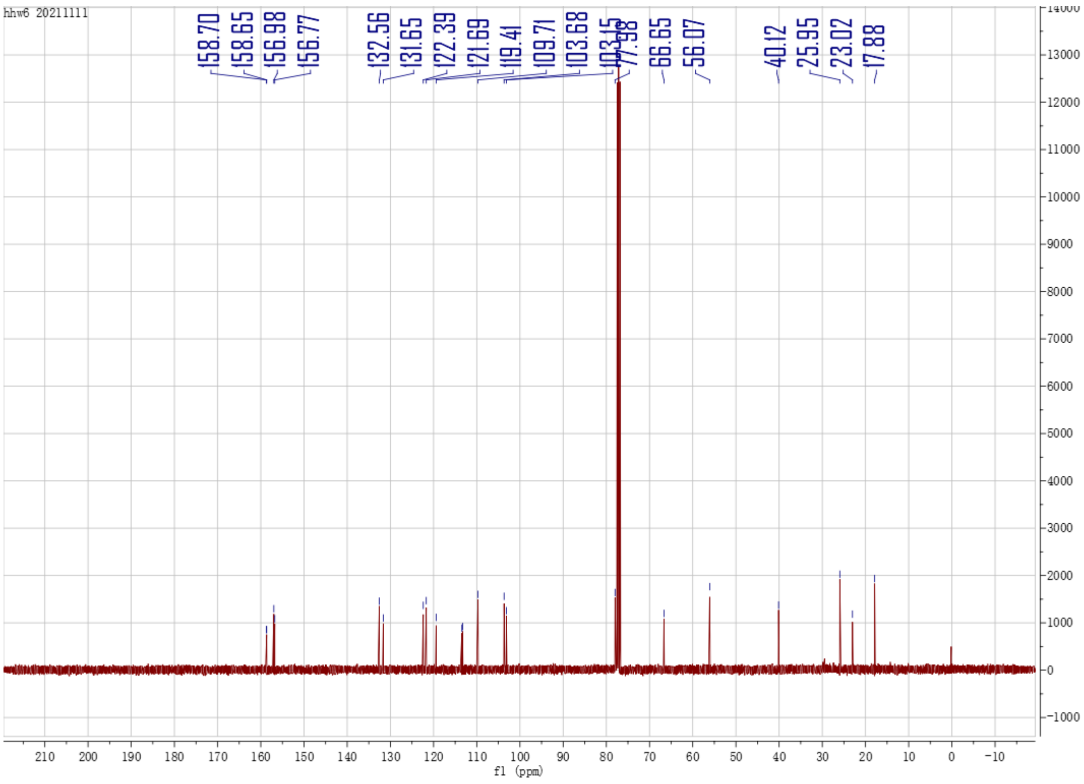

Compound 5 ^{13}C-NMR

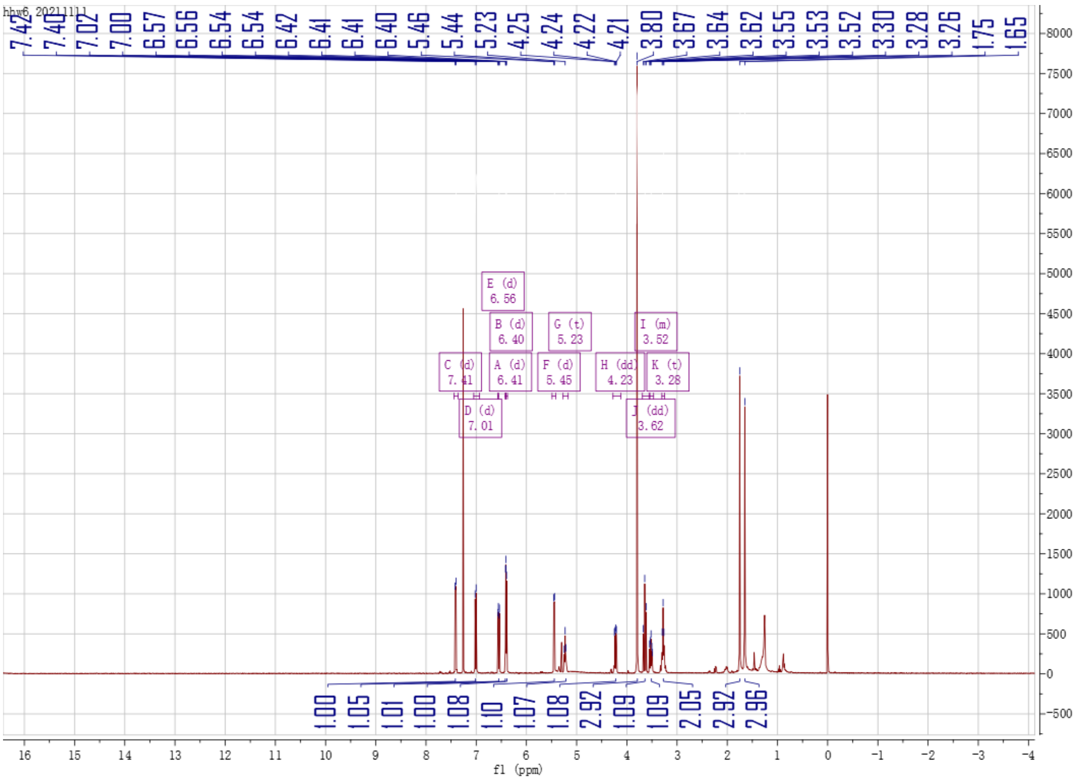

Compound 5 ^{1}H-NMR

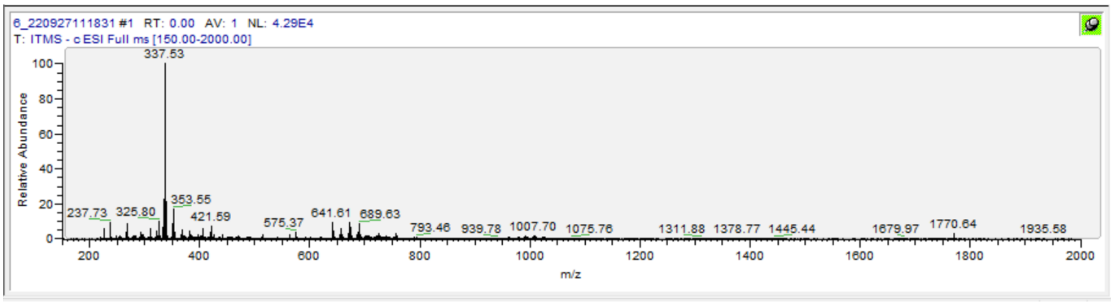

Compound 5 M/Z

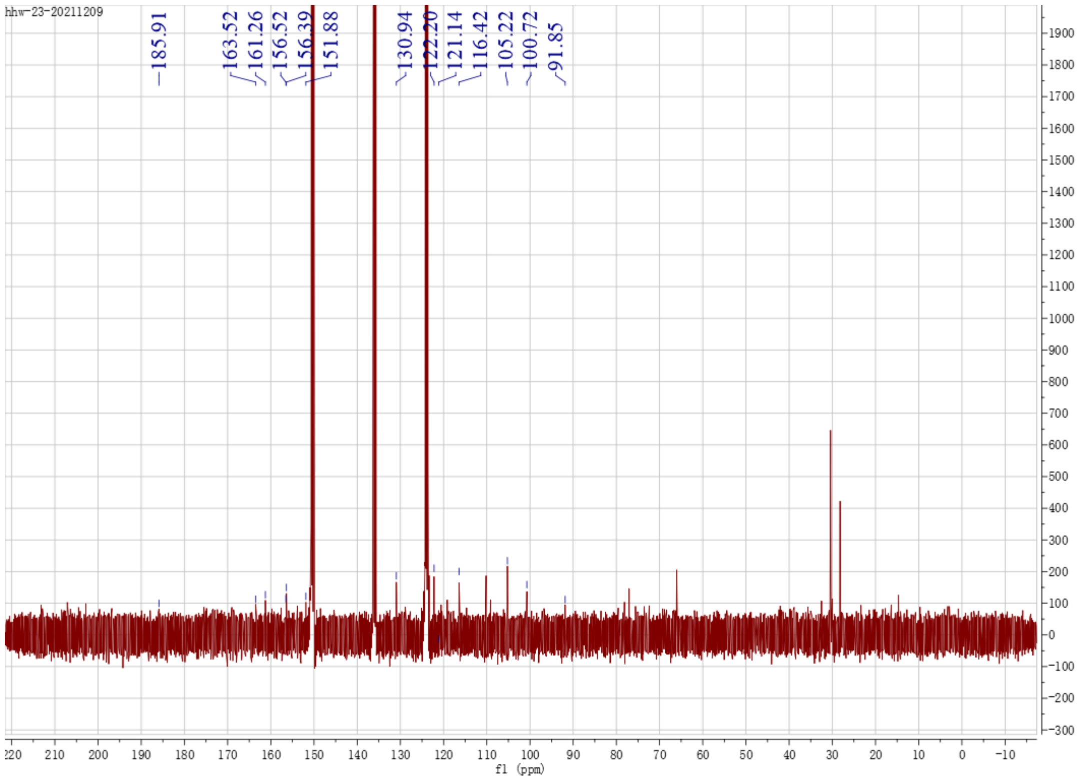

Compound 6 ^{13}C-NMR

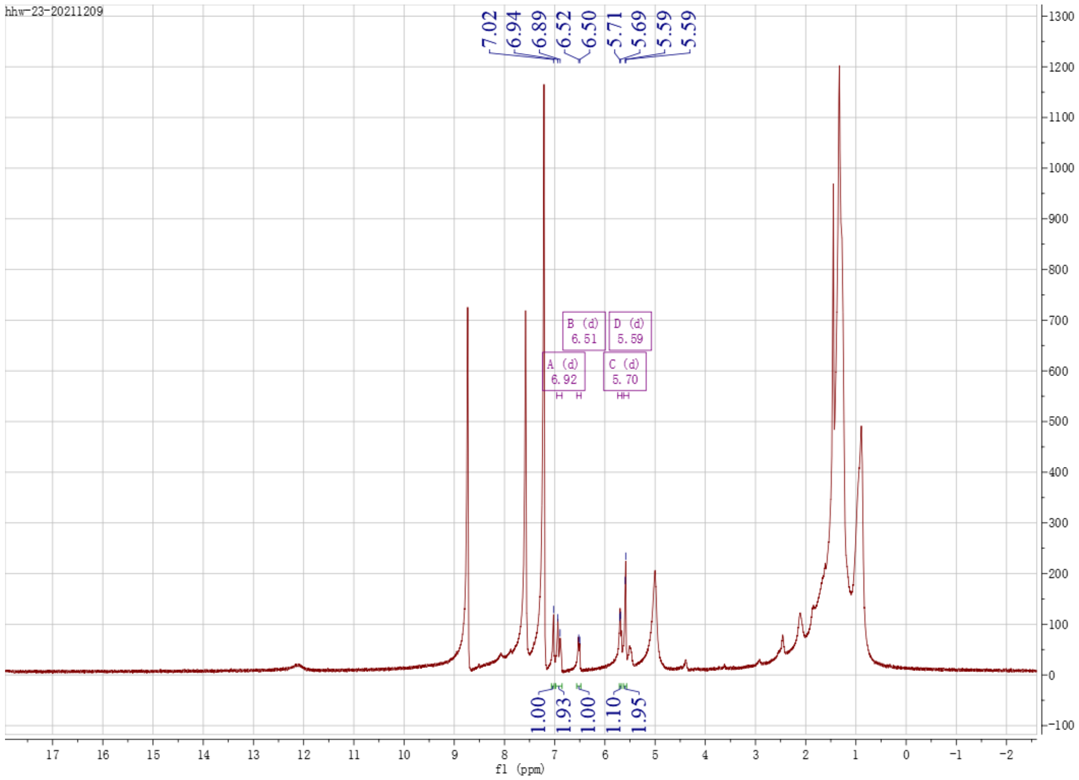

Compound 6 ^{1}H-NMR

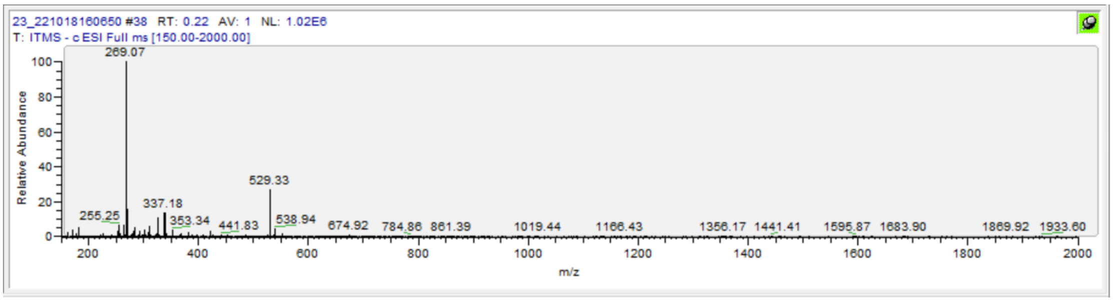

Compound 6 M/Z

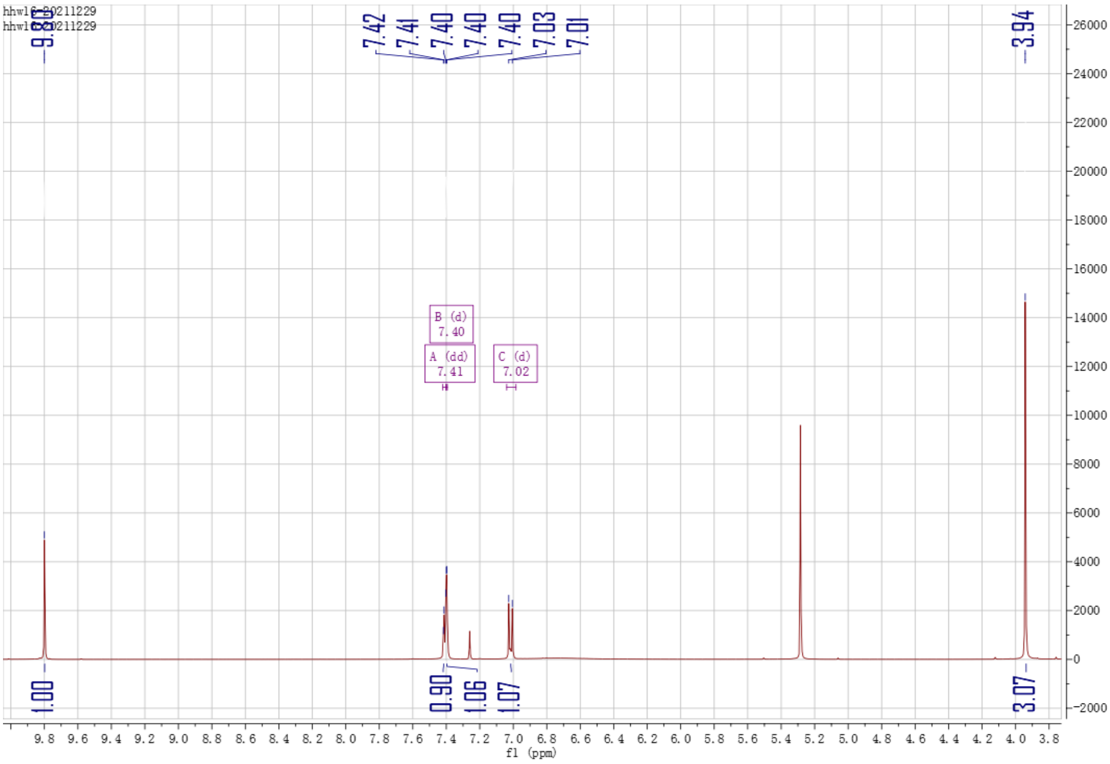

Compound 7 ^{1}H-NMR

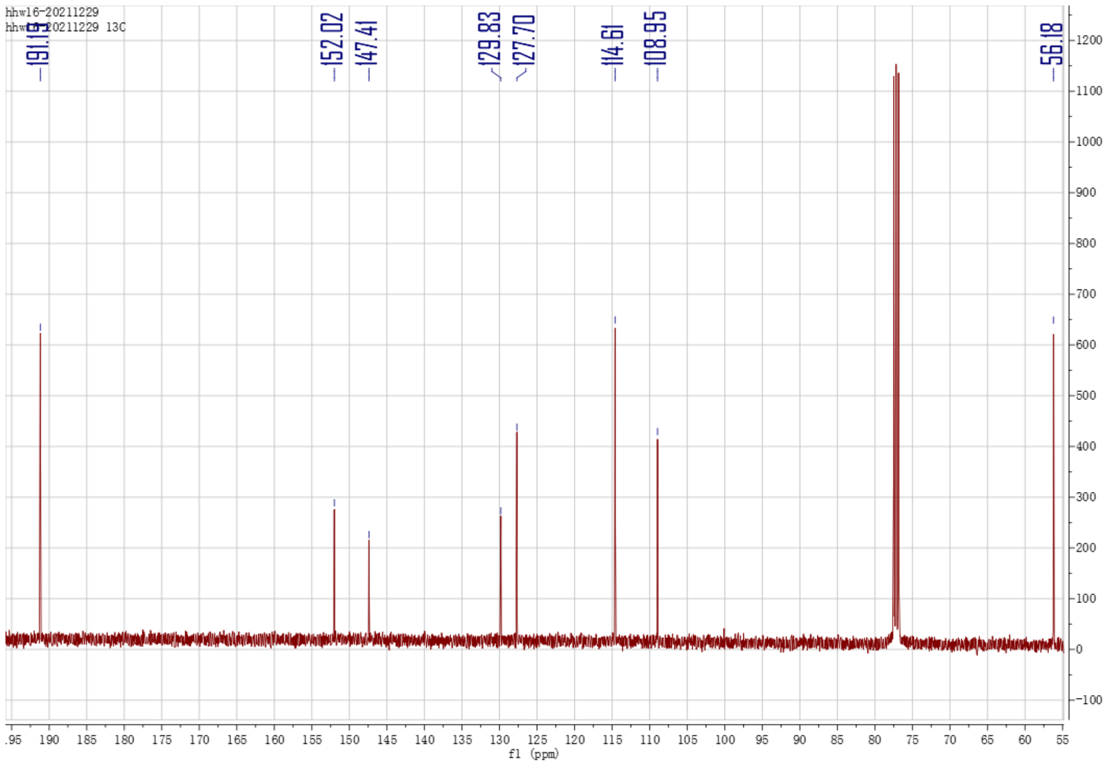

Compound 7 ^{13}C-NMR

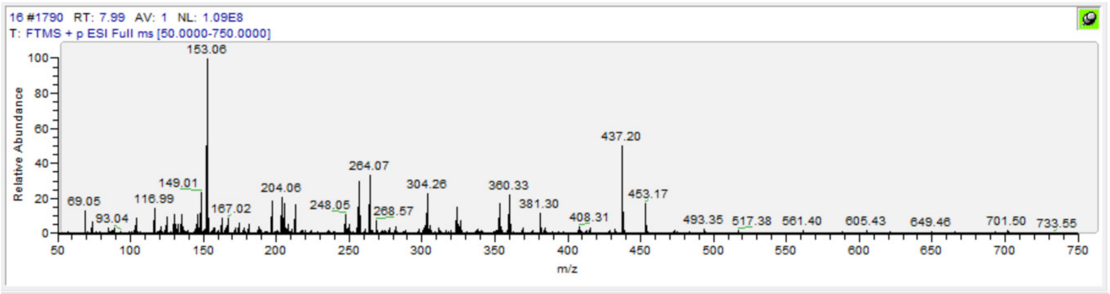

Compound 7 M/Z

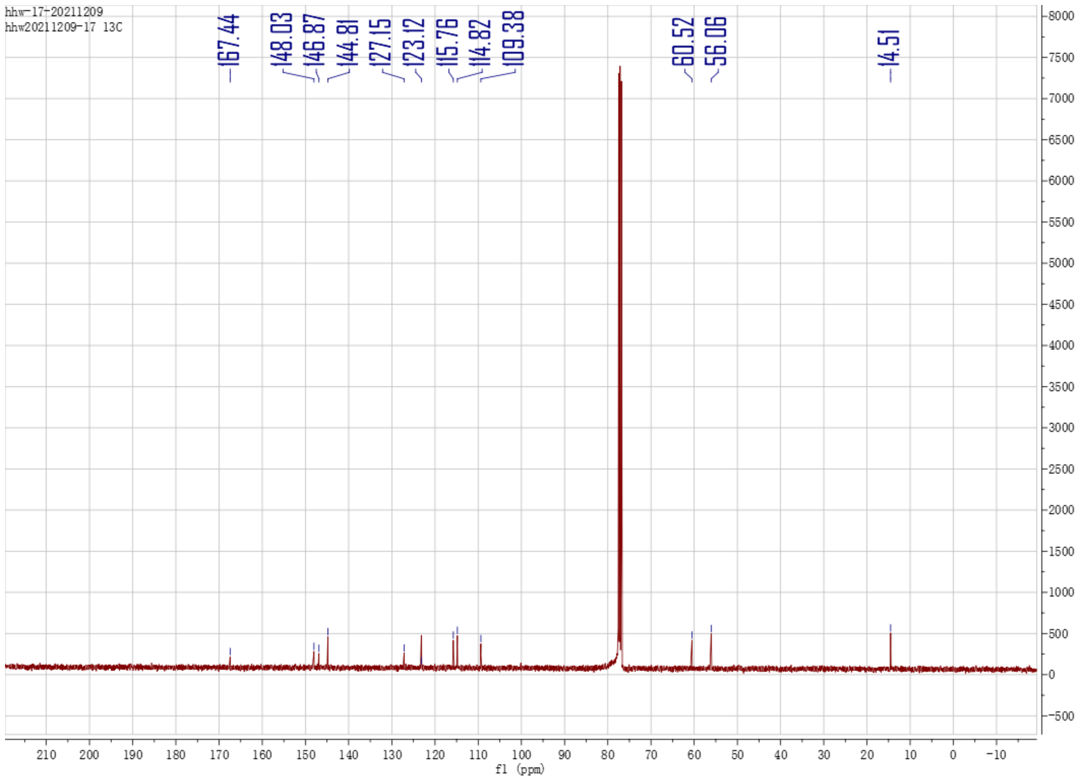

Compound 8 ^{13}C-NMR

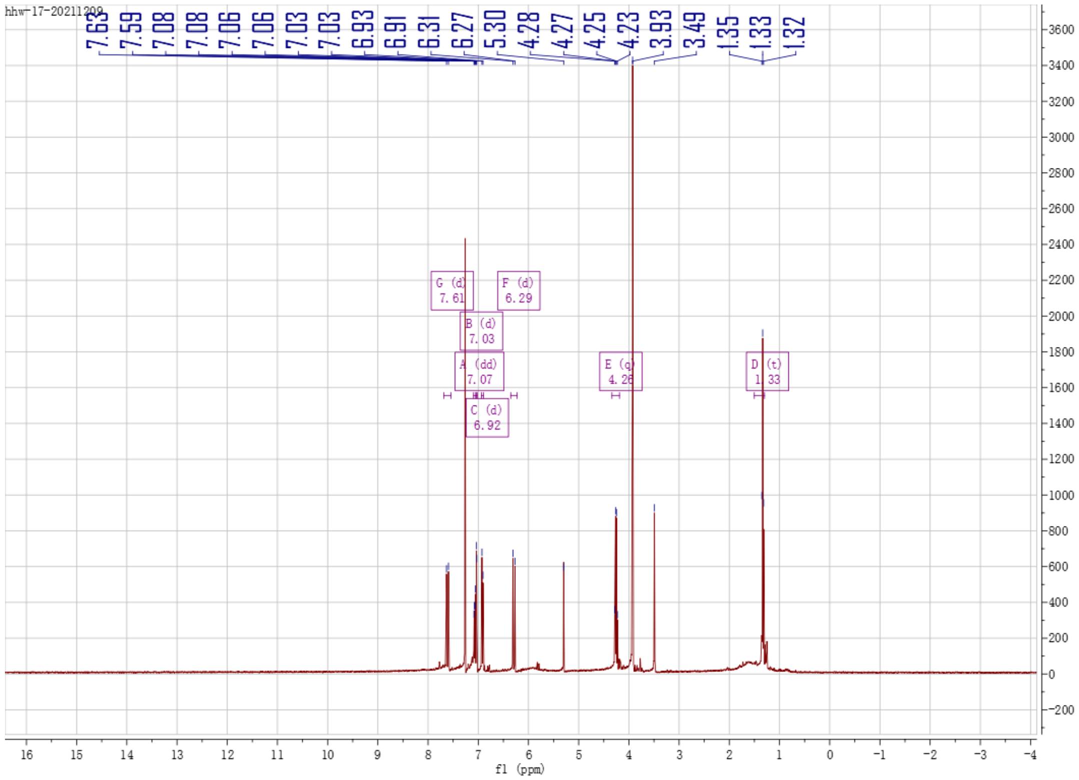

Compound 8 ^{1}H-NMR

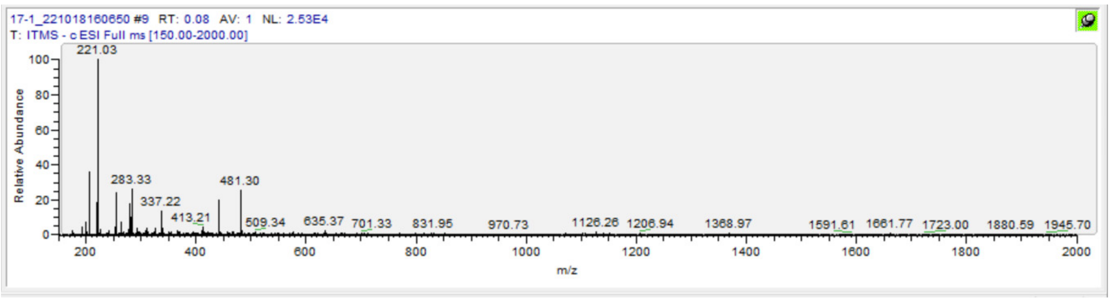

Compound 8 M/Z

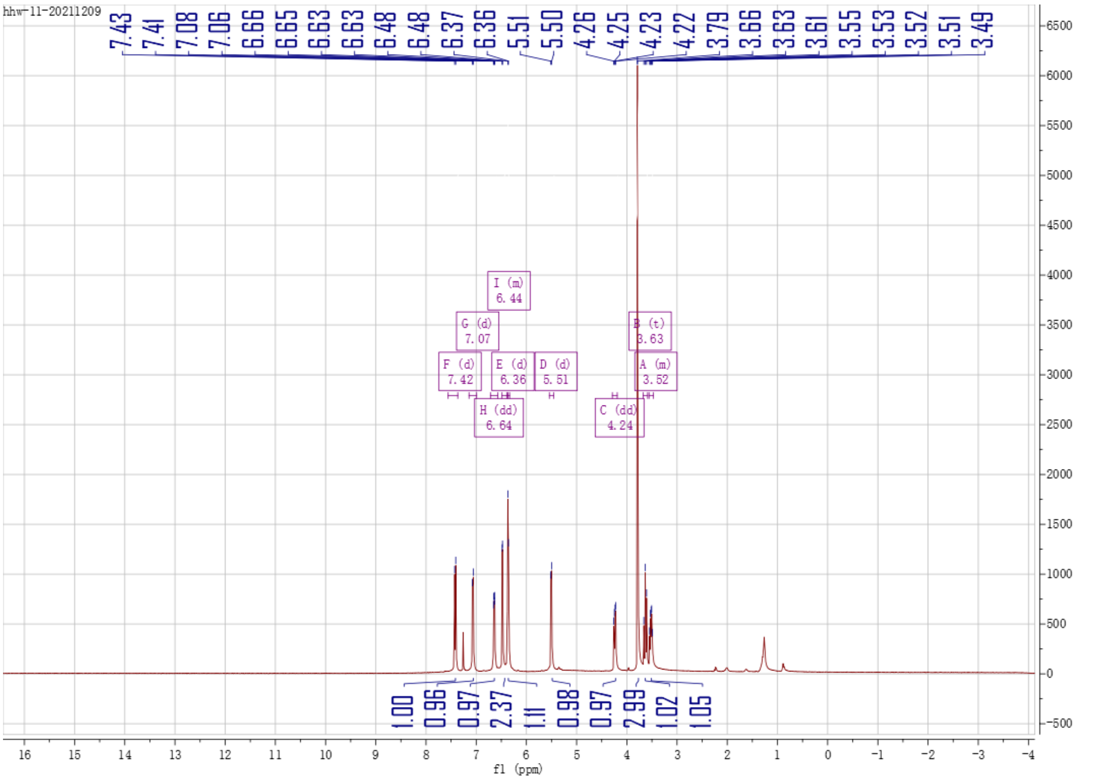

Compound 9 ^{1}H-NMR

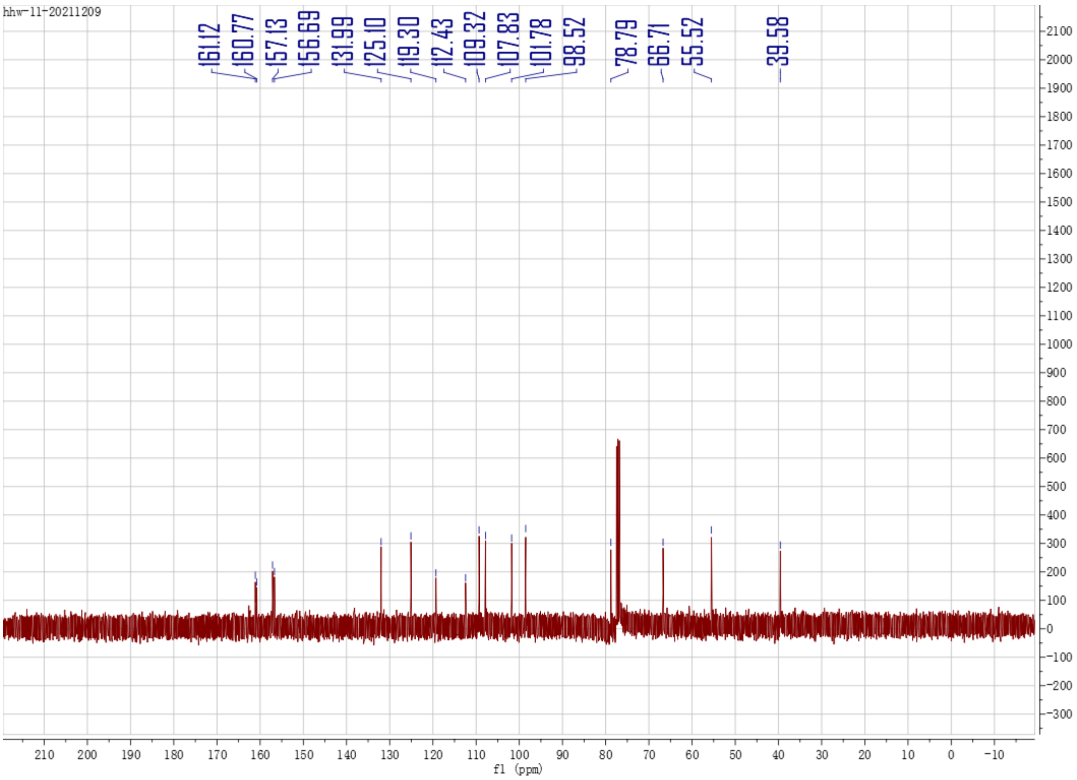

Compound 9 ^{13}C-NMR

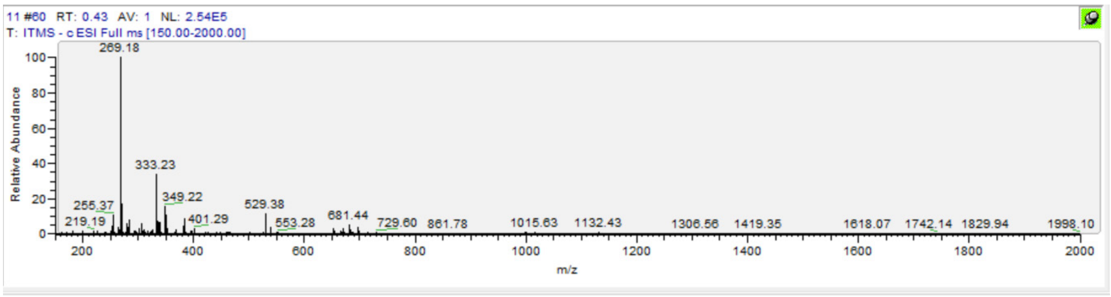

Compound 9 M/Z

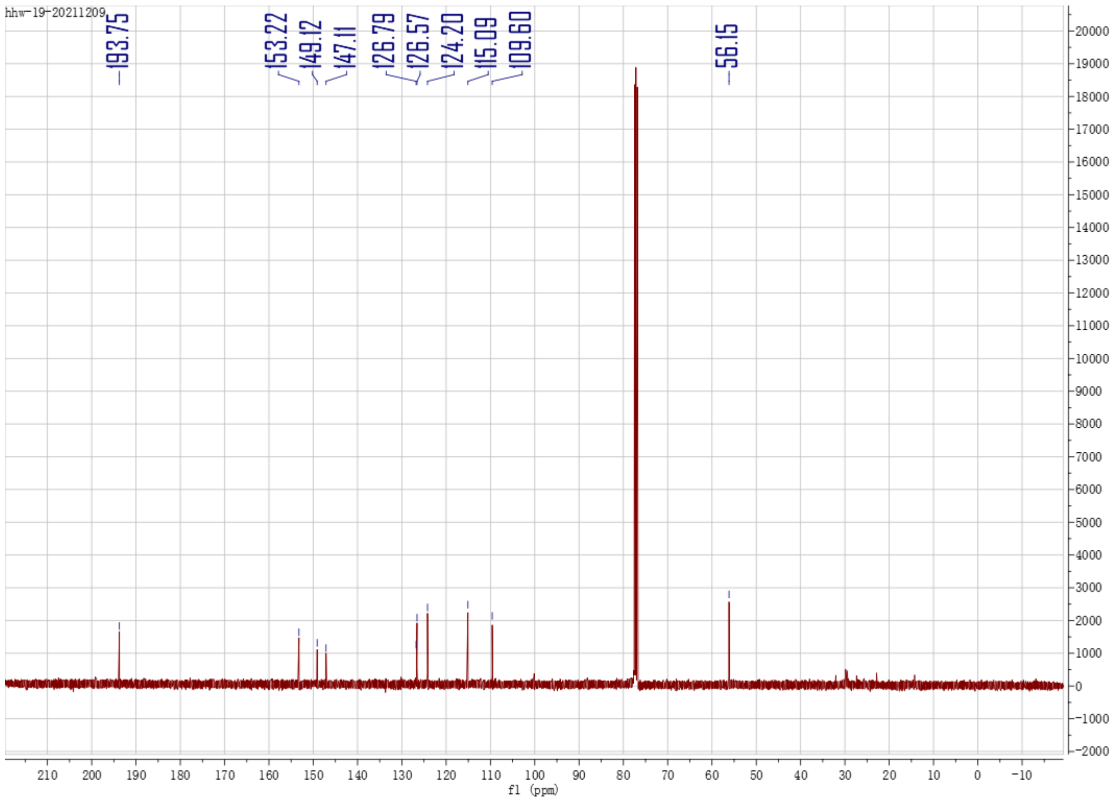

Compound 10 ^{13}C-NMR

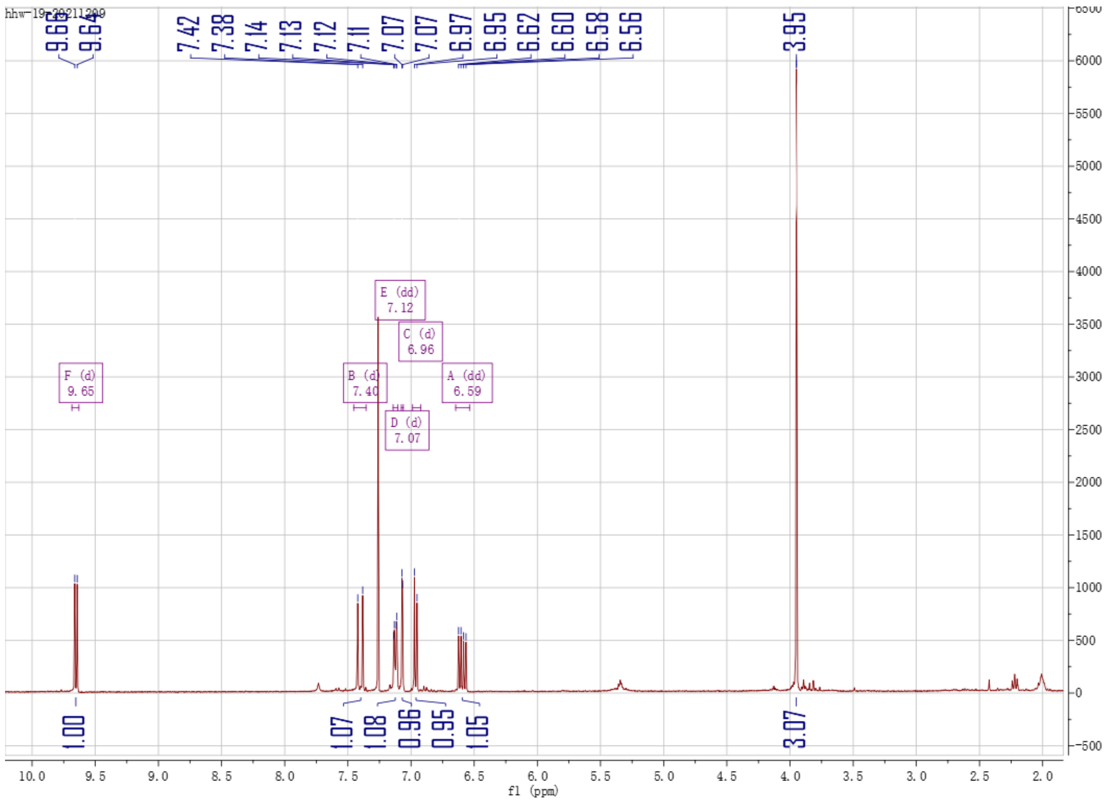

Compound 10 ^{1}H-NMR

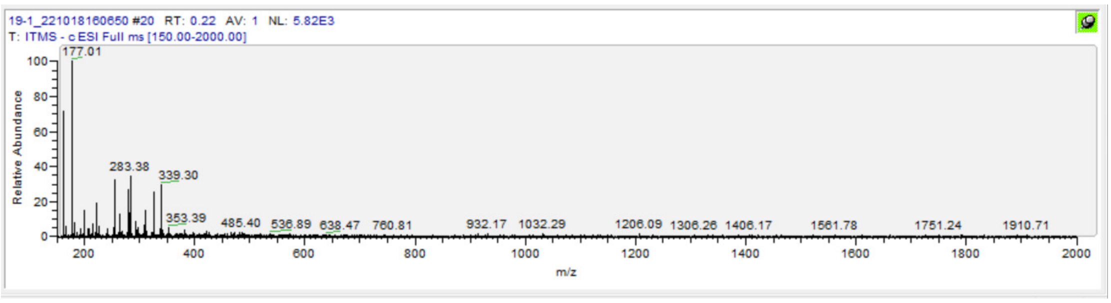

Compound 10 M/Z

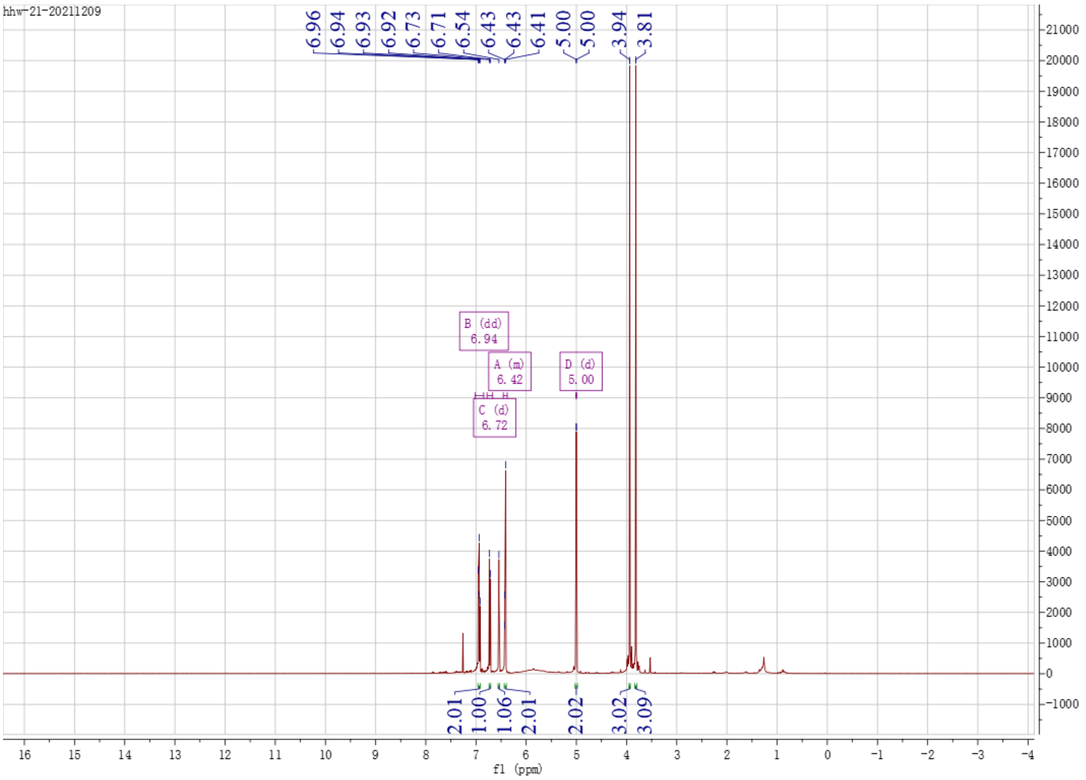

Compound 11 ^{1}H-NMR

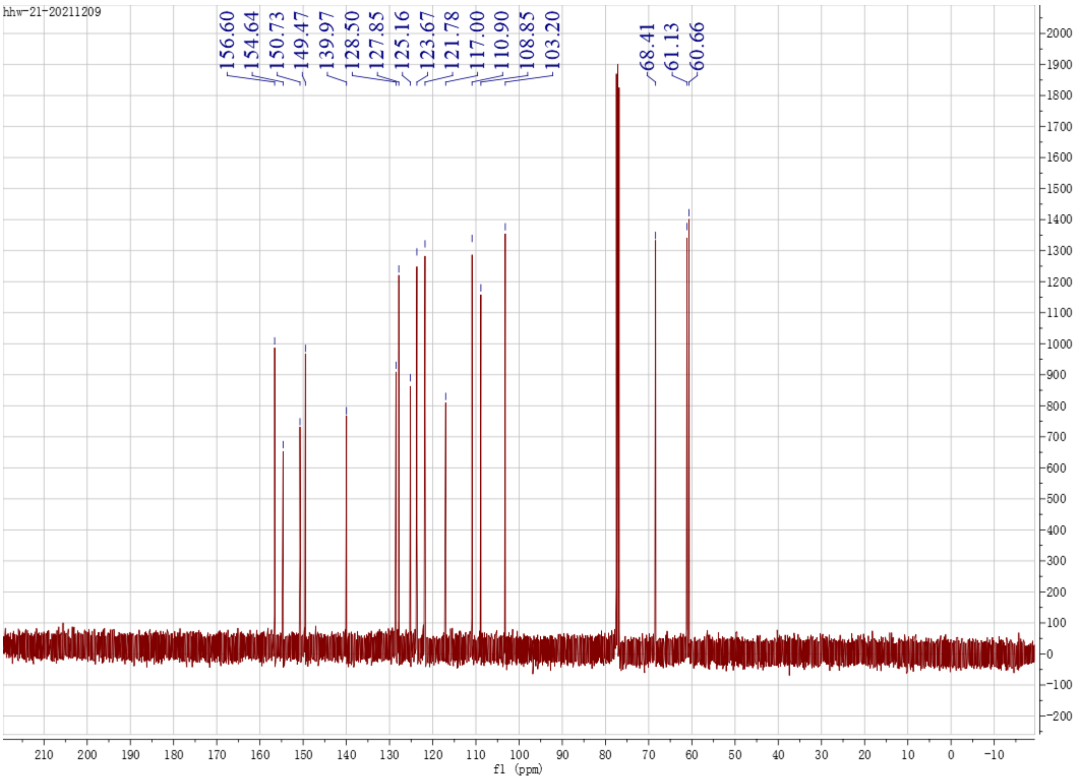

Compound 11 ^{13}C-NMR

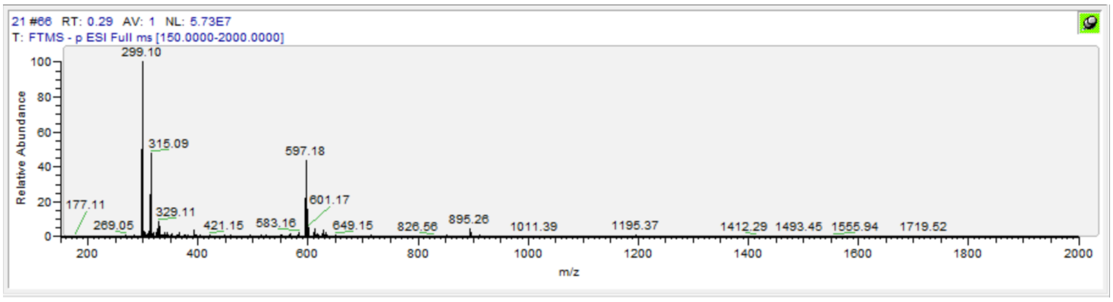

Compound 11 M/Z

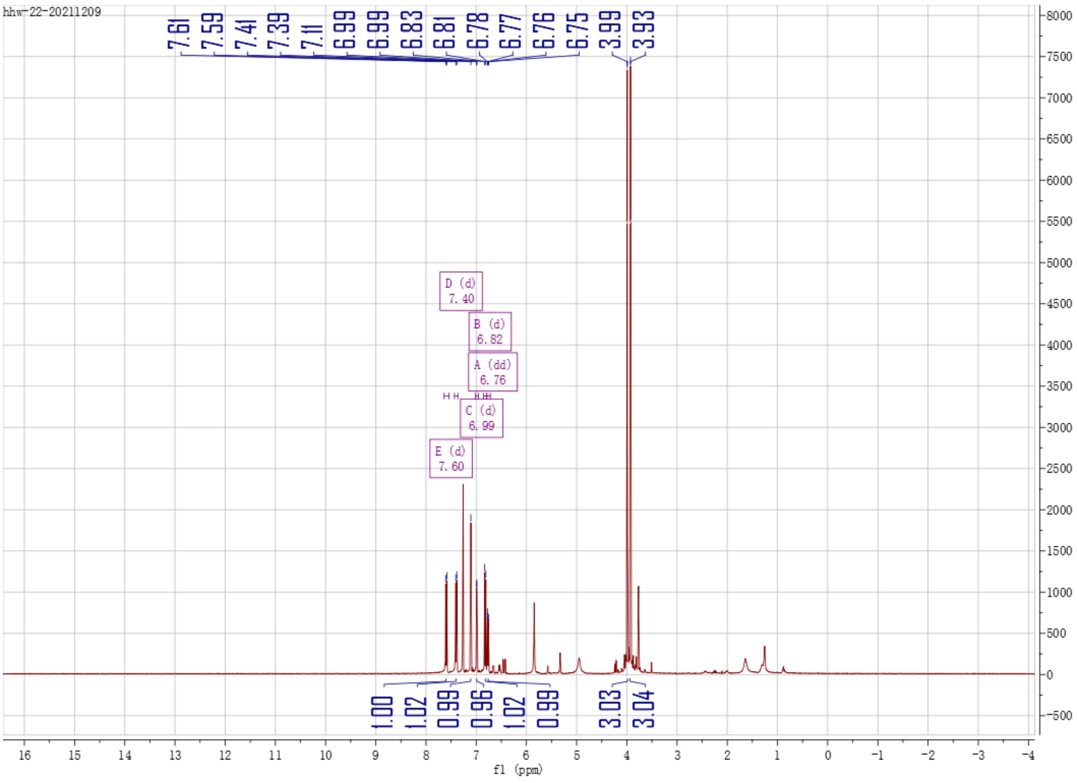

Compound 12 ^{1}H-NMR

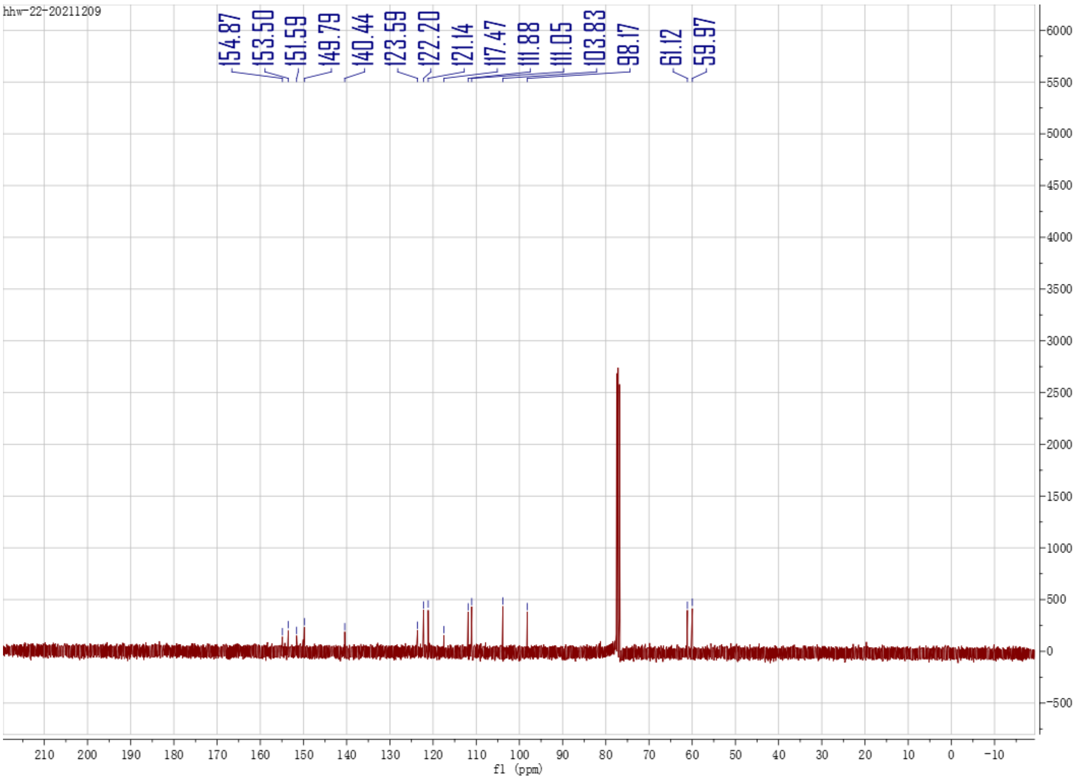

Compound 12 ^{13}C-NMR

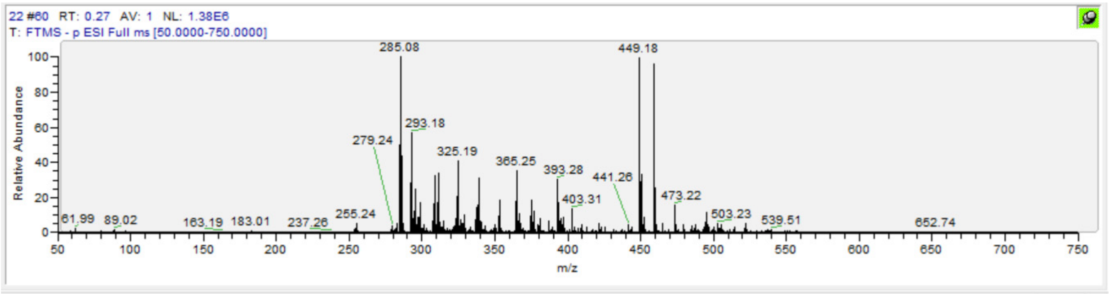

Compound 12 M/Z

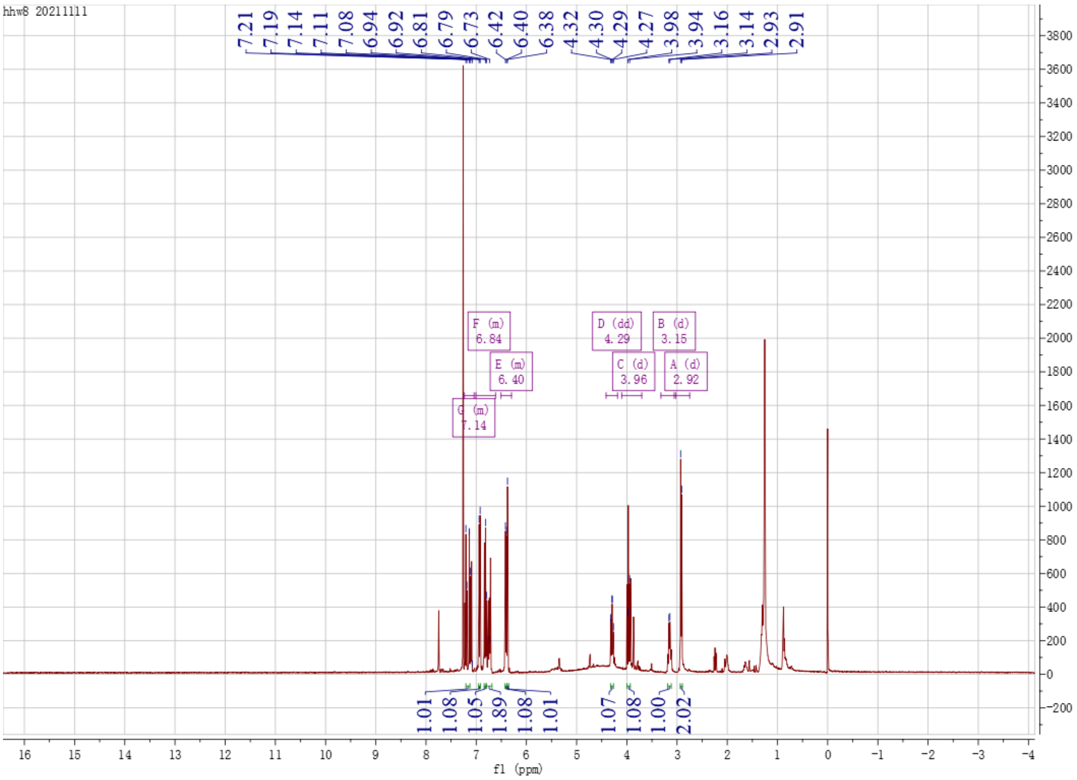

Compound 13 ^{1}H-NMR

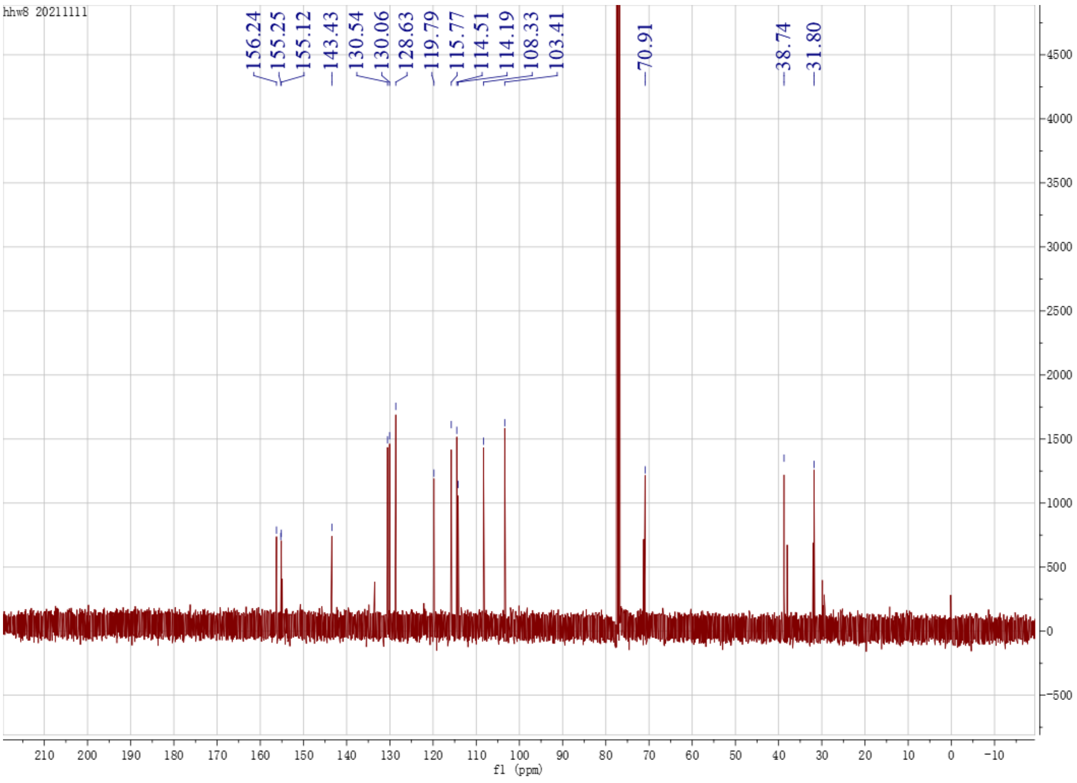

Compound 13 ^{13}C-NMR

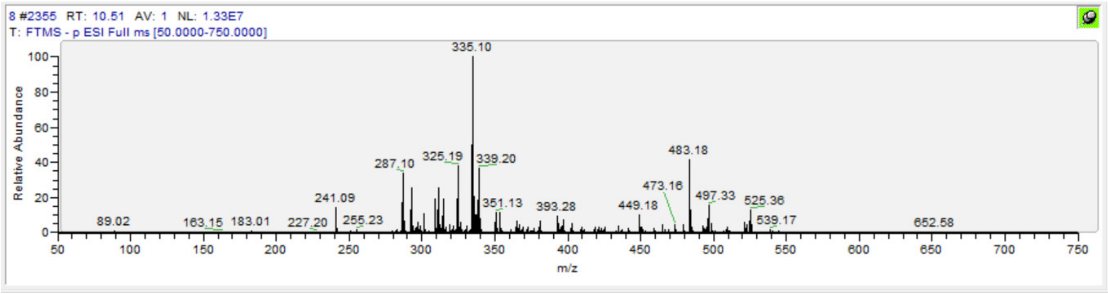

Compound 13 M/Z

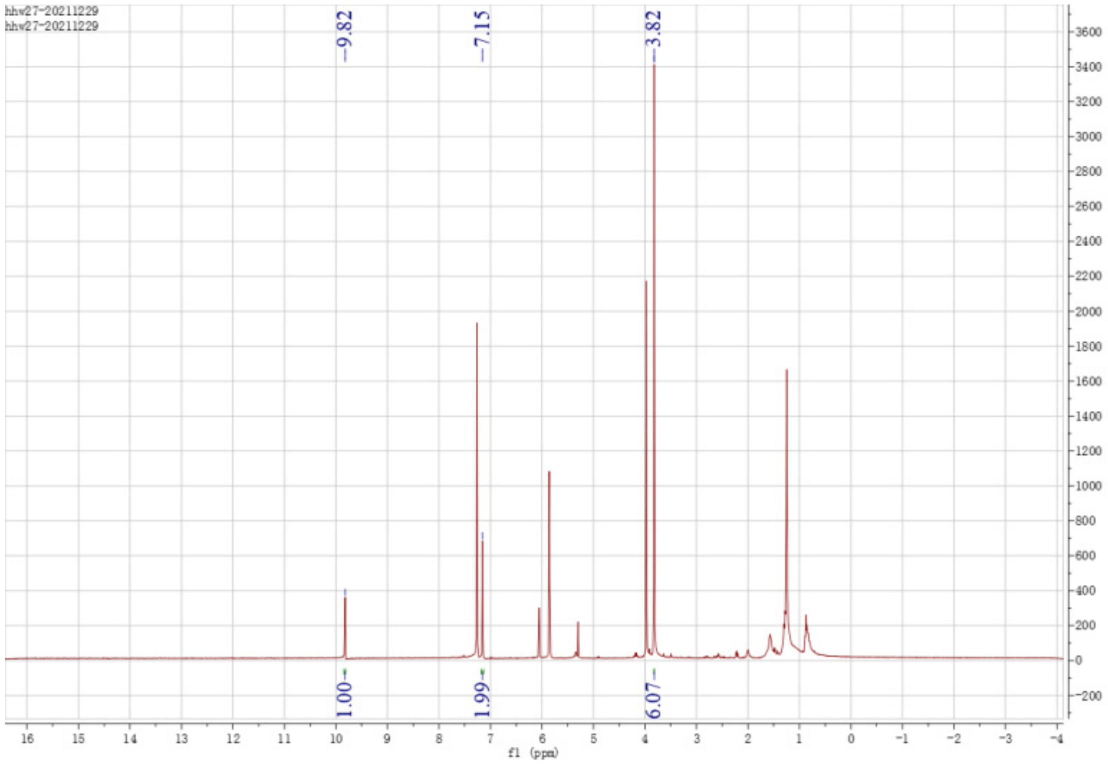

Compound 14 ^{1}H-NMR

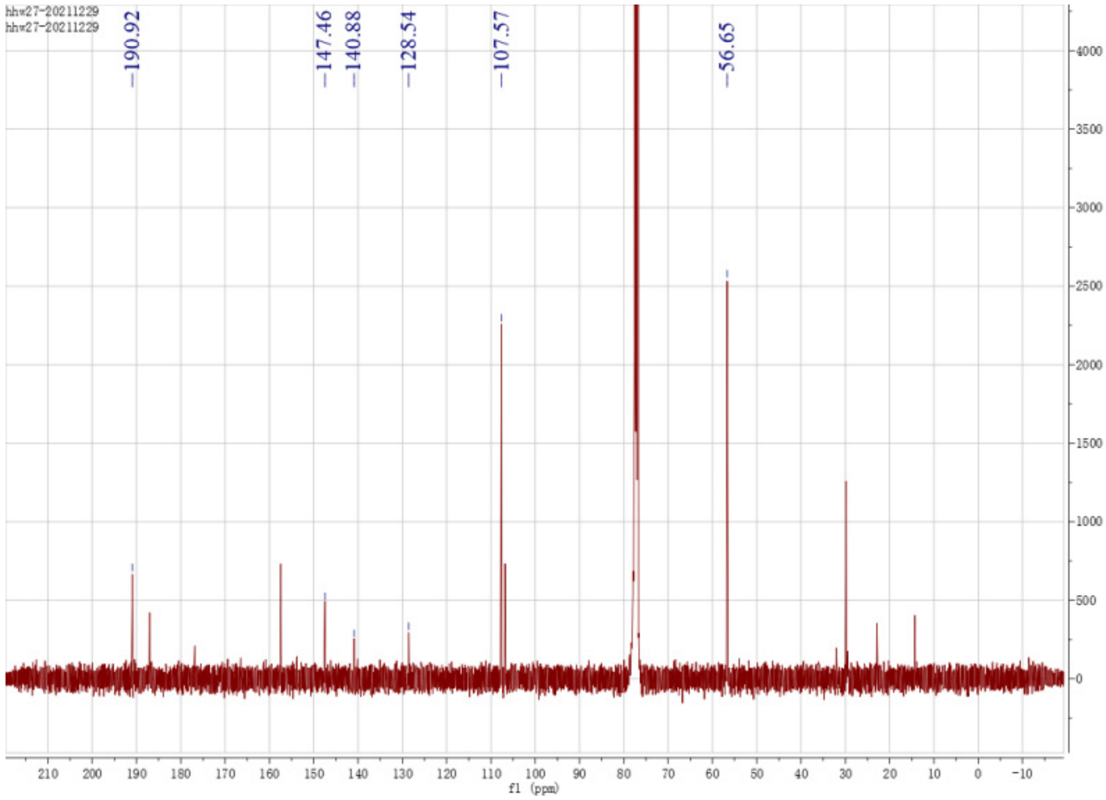

Compound 14 ^{13}C-NMR

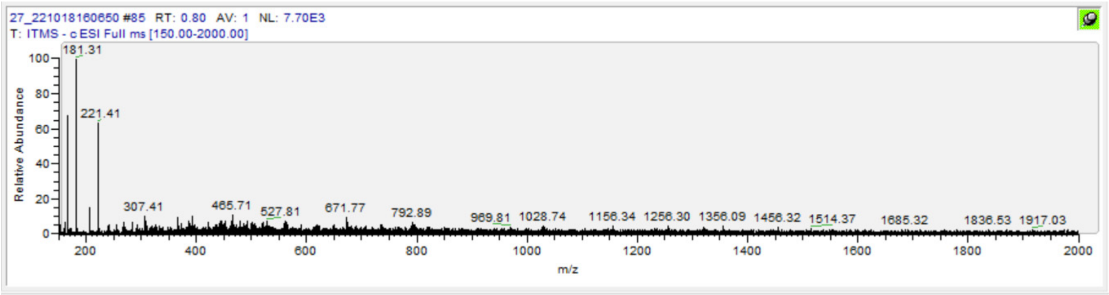

Compound 14 M/Z
